# Dietary intake alters behavioral recovery and gene expression profiles in the brain of juvenile rats that have experienced a concussion

**DOI:** 10.3389/fnbeh.2015.00017

**Published:** 2015-02-05

**Authors:** Richelle Mychasiuk, Harleen Hehar, Irene Ma, Michael J. Esser

**Affiliations:** Faculty of Medicine, Alberta Children's Hospital Research Institute, University of CalgaryCalgary, AB, Canada

**Keywords:** caloric restriction, high fat diet, mild traumatic brain injury, qRT-PCR, telomere, sex-differences

## Abstract

Concussion and mild traumatic brain injury (mTBI) research has made minimal progress diagnosing who will suffer from lingering symptomology or generating effective treatment strategies. Research demonstrates that dietary intake affects many biological systems including brain and neurological health. This study determined if exposure to a high fat diet (HFD) or caloric restriction (CR) altered post-concussion susceptibility or resiliency using a rodent model of pediatric concussion. Rats were maintained on HFD, CR, or standard diet (STD) throughout life (including the prenatal period and weaning). At postnatal day 30, male and female rats experienced a concussion or a sham injury which was followed by 17 days of testing. Prefrontal cortex and hippocampus tissue was collected for molecular profiling. Gene expression changes in *BDNF*, *CREB*, *DNMT1*, *FGF-2*, *IGF1*, *LEP*, *PGC-1α*, *SIRT1*, *Tau*, and *TERT* were analyzed with respect to injury and diet. Analysis of telomere length (TL) using peripheral skin cells and brain tissue found that TL in skin significantly correlated with TL in brain tissue and TL was affected by dietary intake and injury status. With respect to mTBI outcomes, diet was correlated with recovery as animals on the HFD often displayed poorer performance than animals on the CR diet. Molecular analysis demonstrated that diet induced epigenetic changes that can be associated with differences in individual predisposition and resiliency to post-concussion syndrome.

## Introduction

Pediatric concussion and mild traumatic brain injury (mTBI) are three times more common than other brain injuries with 10% of children expected to experience a concussion before the age of 10 years (Barlow et al., 2010; DeWitt et al., 2013). Although it is considered a mild injury, the initial symptoms can be very distressing and a significant proportion of children may suffer from lingering or permanent impairment (Barlow et al., 2010). Current research has focused on treatment strategies after the injury, rather than the factors that predict differential susceptibility or resilience to outcomes. In general, children demonstrate a great degree of variability in their individual susceptibility or resilience to poor outcomes after concussion. Unfortunately there are currently no prognostic tests that are able to differentiate how a child is likely to do after the concussion (McCrory et al., 2013), making it difficult to discern those who would benefit from early access to additional support. Many recent studies have demonstrated that dietary intake is linked to cognitive abilities, brain evolution, and neurological health (for review see Gomez-Pinilla, 2008). As dietary has well appreciated effects on molecular systems and the maintenance of cognitive function, it seems reasonable to consider whether diet also affects outcomes following an mTBI.

There is increasing evidence establishing a link between obesity and adverse neurological outcomes such as reduced cognitive functioning, stroke, and Alzheimer's Disease (Luchsinger et al., 2002). While the exact pathophysiological mechanisms of this association is unknown, excessive caloric intake is believed to directly modulate brain plasticity (Weindruch and Sohal, 1997; Garrido, 2011). Recent animal studies have indicated that high fat diets (HFD) are associated with impaired learning, reduced hippocampal dendritic spine density, and reduced long-term potentiation (Stranahan et al., 2008). Furthermore, HFDs have also been associated with increased inflammation in the brain (Pistell et al., 2010) and impaired hippocampal neurogenesis (Lindqvist et al., 2006). In addition, studies in humans demonstrate a relationship between impaired neuroplasticity and obesity. Indeed, obesity in humans has been shown to impair recovery from stroke (Kalichman et al., 2007), is a major risk factor for ischemic stroke across all ethnic groups, and is associated with the greatest risk of stroke in younger individuals (Suk et al., 2003). Research also indicates that obese individuals have more complications and higher mortality rates following hospitalization for TBI (Brown et al., 2006). What's more, recent statistics released by the World Health Organization indicate that in industrialized countries like the United States and Canada, over 60% of the population is at risk for being overweight (as determined with a BMI ≥25) (https://apps.who.int/infobase).

Conversely, dietary restriction and lowered caloric intake have been associated with increased synaptic plasticity, reduced neurodegeneration, improved brain health, and increased life span (see Masoro, 2005 for review). Human studies and studies using animal models have demonstrated that caloric restriction (CR) protects the brain from neurodegenerative processes commonly seen in Alzheimer's, Huntington's and Parkinson's disease (e.g., Luchsinger et al., 2002; Mattson, 2000, 2010; Mattson et al., 2003; Wang et al., 2005.) CR has been shown to increase neurogenesis in the adult brain (Lee et al., 2002), prevent age-related declines in LTP (Eckless-Smith et al., 2000) and improve cognitive function, and memory performance in the elderly (Witte et al., 2009). Although the link between CR and neurological function is not fully understood, studies suggest that CR may be related to reduced oxidative stress, regulation of metabolic pathways and increased genomic stability (for review see Li et al., 2011). Interestingly, studies involving moderate TBI have demonstrated that CR prior to or immediately after an injury was neuroprotective; reducing tissue damage and improving post-injury cognitive function (Davis et al., 2008; Rich et al., 2010). As chronic CR has been shown to be neuroprotective, and long-term exposure to diets high in fat have been associated with adverse neurological outcomes, variations in dietary intake could be significant predictors of differential susceptibility to resilient or poor outcomes following concussion early in life.

This study sought to determine if chronic exposure (including the perinatal period) to a HFD or CR would alter susceptibility or resiliency to poor outcomes following an mTBI using an animal model of pediatric concussion. The study examined three distinct post-injury outcome measures; (1) a behavioral test battery including measures of balance and motor coordination (beam-walking and open field), emotional responsivity (elevated plus maze), and cognitive functioning (novel context mismatch and Morris water task); this behavioral test battery has been previously used in this laboratory and represents a comprehensive measurement of symptoms that closely represent pediatric presentation of post-concussion syndrome. (2) A molecular profile that included expression level changes in the prefrontal cortex (PFC) and hippocampus (HPC) of genes involved in regulating dietary-dependent changes in neuroplasticity and healthy neurodevelopment (*BDNF*, *CREB*, *DNMT1*, *FGF-2*, *IGF1*, *Leptin*, *PGC-1α*, *SIRT1*, *Tau*, and *TERT*); these two brain regions have been linked to many of the functional deficits associated with post-concussion syndrome, and the genes selected encompass a range of factors important for normal brain development, neuroplasticity and recovery from injury, in addition to long-term neurological health with respect to dietary intake. (3) Telomere length (TL). Determination of TL from peripheral skin cells, the PFC, and the HPC was obtained in effort to generate a predictive biomarker that could be used to help determine those at risk for poor outcomes following mTBI. As a variety of environmental factors (including diet) have been shown to alter the rate of telomere shortening (Valdes et al., 2005), and short telomeres are associated with premature aging and neurodegeneration (Blasco, 2005), telomere length may provide researchers and clinicians with a useful prognostic tool for the treatment of mTBI.

## Materials and methods

### Breeding and dietary procedure

All experiments were carried out in accordance with the Canadian Council of Animal Care and approved by the University of Calgary Conjoint Faculties Research Ethics Approval board. All animals were maintained on a 12:12 hr light:dark cycle (lights on at 0700) in a temperature controlled breeding room (21°C). Fourteen Sprague Dawley female rats were pair-housed (2 female rats/cage) in standard shoe-box cages. Six female rats had *ad libitum* access to standard food and water (STD), four female rats had *ad libitum* access to a high fat, high sugar diet (HFD), and the remaining four female rats were maintained on a calorically restricted diet of standard rat chow with *ad libitum* access to water (CR). The HFD was an adjusted calories diet whereby 60% of the rat's total calories were derived from fat, (TD.06414, Harlan Laboratories—Madison, WI) which was supplemented with drinking water that contained 20% sucrose. Studies using a HFD similar to ours demonstrated increased serum cholesterol, elevated fasting blood glucose levels, but no changes in serum insulin levels (Stranahan et al., 2008). A brief description of the differences between the HFD and SD are as follows: calories from fat HFD: 60.3%, SD: 12.14%; calories from protein, HFD: 18.4%, SD: 28.76%; calories from carbohydrates, HFD: 21.3%, SD: 59.06%; potassium, HFD: 1.28%, SD: 1.20%; Sodium, HFD: 0.36%, SD: 0.40%; Magnesium, HFD: 0.18%, SD: 0.24%; Vitamin A, HFD: 16.1 ppm, SD: 21.0 ppm; Vitamin D3, HFD: 4.2 ppm, SD: 5.0 ppm; and Riboflavin, HFD: 12.6 ppm, SD: 12.0 ppm (Harlan Laboratories, Diet # TD.06414, and RMH2500). The CR diet used the standard rat chow provided to the control animals, but restricted access to the food using an alternate day feeding regime (rats had *ad libitum* access to the food every other day). Diets that follow this regime have been shown to reduce caloric intake by roughly 40% (Sharma and Kaur, 2005), in addition to increasing lifespan, improving neuroplasticity, and reducing disease manifestation (for review see, Masoro, 2005). Female rats began their respective diets 3 weeks prior to mating to ensure that the females had adjusted to the dietary manipulation before breeding. Each female rat was removed from the pair-house to be mated with an individual male rat that had been fed the standard diet. Following mating, the female rats were returned to their female cage-mate where they remained until the day prior to delivery. At this point, female rats were separated and remained individually housed with their litters for the duration of weaning. Thus, female rats were maintained on their respective diets for a total of 9 weeks; 3 weeks prior to mating, the 3 weeks of pregnancy, and throughout weaning. Pups stayed on the same diet as their mothers even after weaning; i.e., pups born to mothers on the HFD continued to consume the HFD for the duration of testing. This diet regime was continued after weaning because literature shows that children and young adults are highly influenced by parental eating habits and are likely to maintain dietary habits similar to those learned in the family environment (Hood et al., 2000; Savage et al., 2007).

Dams were weighed daily prior to, as well as, during pregnancy, and for the duration of weaning to ensure that the dietary manipulations were not placing the dams or pups at risk. Following weaning and for the duration of the experiment, pups were weighed every other day. The 4 HFD mothers gave birth to 52 pups (26M: 26F), the 4 CR mothers gave birth to 48 pups (28M: 20F) and the 6 STD mothers gave birth to 72 pups (35M: 37F). Pups were weaned from their mothers at postnatal day 21 (P21) and randomly assigned to receive a mTBI or sham injury. To prevent litter effects, randomization was carried out so that the cage held four pups of the same-sex and same dietary exposure, but derived from different mothers. Seventy-two pups were used in this experiment (24 HFD, 24 CR, and 24 STD), with the remaining pups being used in a different study published elsewhere. There was no difference in average animal body weight at the time of injury or at the time of sacrifice for any of the dietary groups (data not shown).

### mTBI procedure

When pups reached P30, half of the animals received a mTBI using a modified weight-drop technique (Mychasiuk et al., 2014a) and the other half received a sham injury. Briefly, animals were lightly anesthetized with isoflourane and placed chest down on a scored piece of tinfoil that is suspended 10 cm above a foam collection sponge. To produce the mTBI, a 150 g is dropped through a guide tube and produces a glancing impact to the closed skull of the rat. The impact from the weight propels the rat through the scored tinfoil, where it undergoes a 180° vertical rotation before landing on its back on the collection sponge. Immediately after the impact, topical lidocaine is applied to the rat's head and it is placed in a clean warm cage to recover. Sham animals that do not receive an injury are lightly anesthetized, placed chest down on the scored tinfoil but the removed before the weight is dropped, receive topical lidocaine and are also placed in a clean warm cage to recover. The time it took each rat to right itself (flip from the supine position to a prone/standing position) in the recovery cage was recorded as the *time-to right*. Each cage of 4 pups was designed to contain 2 mTBI and 2 sham animals. Following the injury pups were given 24 h to recover which was followed by 15 consecutive days of behavioral testing.

### Behavioral testing

#### Beam walking

At P31 (24 h post-mTBI) animals were tested in a beam-walking paradigm similar to that described by Schallert et al. (2002). Rats were required to traverse a 165 cm long tapered beam with “safety ledges” (2 cm wide) that was suspended 1 meter in the air. The rats started at the wide end of the tapered beam and walked toward the narrow end. The rat's home cage is placed at the end of the beam and acts as a reinforcement cue for task completion. The rat is provided a single trial to learn to walk from the start point to the home-cage. Once the rat reaches the home-cage it is permitted to remain in the cage for 1 min to support target location. The rat is given four additional videotaped trials, each separated by 1 min rest periods. The video camera is set up at the start point and is positioned to view down the length of the tapered beam. A trained research associate blinded to the experimental groups scored the videos for the number of hind leg foot-slips. A foot-slip is identified as any time one of the rat's hind feet uses the safety ledge while walking the length of the beam.

#### Open field

At P32 (48 h post-mTBI) rats were tested in the open field task. Briefly, rats are placed in the center of a circular open field (diameter 100 cm) and allowed to explore the environment for 10 min. An overhead camera was used to track the rat's overall movement (distance traveled and speed of travel). The open field was cleaned with Virkon® between each testing session.

#### Elevated plus maze (EPM)

On P33, rats were tested in the EPM. The EPM was constructed of black Plexiglas® and contained two open arms and two closed arms that intersect in the center. The EPM is situated 55 cm above the ground in a well-lit empty room. A video camera was placed at the end of one of the open arms in a slightly elevated position that allowed visualization of both open arms and the center of the EPM. Rats were placed in the center of the platform with their paws facing one of the closed arms and permitted to explore the maze for 10 min. The EPM was cleaned with Virkon® between each testing session. A research associate blinded to the experimental conditions scored the videos for the time the rats spent in the open arms, closed arms, and center of the EPM.

#### Novel context mismatch (NCM)

From P35-38 rats were tested in the NCM paradigm similar to the protocol described by Spanswick and Sutherland (Spanswick and Sutherland, 2010). Rats were exposed to two distinct contexts (context A and context B) for 5 min each day, context B immediately after context A, for 3 consecutive days. Context A consisted of a clear rectangular box (70 × 40 × 33 cm) with two identical plastic cubes, whereas context B consisted of a dark blue circular bin (diameter 47, 36 cm high) with two identical cylindrical cans. The objects were securely attached to the floor of each context so they could not be moved or knocked over during the exploration period. On the probe day (day 4), rats explored each of the familiar contexts (A and B), one immediately after the other, but were then given a 5 min delay before being permitted to explore a novel context for 5 min. The novel context consisted of either Context A^*modified*^ (with one object from context A and one object from context B) or Context B^*modified*^ (with one object from context B and a second object from context A). Exploration of the novel context was videotaped and a research associate blinded to the experimental conditions scored the amount of time the rats spent with the object in the correct context and with the object that was new to the context. All of the objects and context bins were cleaned with Virkon® between each testing session.

#### Morris water task (MWT)

Using a protocol similar to that described by Sutherland et al. (1988), rats were trained on the MWT from P41-44 and tested in a probe trial on P45. The rats were tested in a circular pool (165 cm diameter, 50 cm high) that was filled with water (22°C). A clear platform was placed in the center of one of the quadrants just below the surface of the water, where it remained for the duration of training. Rats were given 8 trials/day for 4 consecutive days to learn the location of the platform. A learning trial consisted of placing the rat in the water at the perimeter of the pool from ¼ specified locations (north, south, east, or west) and providing it with 60 s to find the hidden platform. The rats learn to use spatial cues in the testing room to find the platform. If a rat failed to find the platform, the experimenter placed the rat on the platform and allotted it 10 s to identify spatial cues that could be used in the next trial. The order of pool entry (north, south, east, or west) was varied from day-to-day to ensure that the rat was not memorizing a specific swim pattern. An overhead camera was used to track the swim speed and route taken by each of the rats. On day 5 (probe trial), the platform was removed from the pool and the rat was placed into the pool from the west location and permitted to swim for 60 s. The overhead camera tracks the rat's pathway, swim speed, and time spent in each of the four pool quadrants.

### Sacrifice and molecular analysis

Once behavioral testing was complete (~P47) rats were sacrificed and brain tissue was used for molecular analysis. Rats were subjected to isoflourane inhalation, weighed and quickly decapitated. Using the Zilles (1985) rat brain atlas for dissection, the prefrontal cortex (PFC) and hippocampus (HPC) were removed and flash frozen on dry ice. The tissue was stored at −80°C.

#### DNA and RNA isolation and quality determination

Using the Allprep RNA/DNA Mini kit (Qiagen, Hilden, Germany), genomic DNA and total RNA was isolated from the frozen brain tissue (PFC and HPC), according to the manufacturers protocol. Concentration and purity of the genomic DNA was measured with Nanodrop 2000 (Thermo Fisher Scientific, Waltham, MA). All sample concentrations were over 100 ng/μl and produced absorbance ratios (A_260/280_) between 1.95 and 2.05. Total RNA concentration and purity was also determined by using the Nanodrop 2000, followed by resolving 1 μg of each sample on 1% agarose gel electrophoresis to determine RNA integrity. Two micrograms of purified total RNA was reverse transcribed to cDNA using oligo(dT)_20_ of the Superscript III First-Strand Synthesis Supermix kit (Invitrogen, Carlsbad, CA), as per manufacturer's protocols.

#### Primer design, quality determination and relative quantitative reverse transcription – polymerase chain reaction (qRT-PCR)

All primers for the target genes and reference genes were purchased from IDT (Coralville, IA). For 6 genes, primers were designed by an in-house technician using Primer3 (http://bioinfo.ut.ee/primer3/) to span exon-exon junctions, whereas for the remaining 8 genes, primers were obtained from previously published results (see Table 1). Expected amplicon size for in-house designed genes was confirmed via gel electrophoresis of the PCR product to ensure there was not DNA amplification of the RNA samples. This was followed with a melt curve analysis using SYBR Green FastMix with Rox (Quanta BioSciences, Gaithersburg, MD) to ensure primer specificity. Gradient PCR was performed to determine the optimal annealing temperature for each primer pair for the target and reference genes.

**Table 1:** **Primer information for relative and absolute qPCR**.

**Gene symbol**	**Gene name**	**Primer sequence**	**Amplicon size (bp)**	**Tm (°C)**	**Cycling parameters**
*Lep*	Leptin	(+)tcaccccattctgagttttgtc	203	60.0	1 cycle 95°C 3 min
		(−)cgccatccaggctctct			50 cycles 95°C 30 sec
					50 cycles Tm°C 1 min
					+Melt Curve
*Bdnf*	Brain-derived neurotrophic factor	(+)ccataaggacgcggacttgt	N/A	60.0	1 cycle 95°C 3 min
		(−)gaggctccaaaggcacttga			40 cycles 95°C 15 sec
*Creb*	cAMP response element binding protein	(+)ccgccagcatgccttc	N/A	60.0	40 cycles Tm°C 30 sec
		(−)tgcagcccaatgaccaaa			+Melt Curve
*Dnmt1*	DNA (cytosine-5-)-methyltransferase 1	(+)agaagagacgaaaaacgacacg	164	60.0	
		(−)cttcaggtcagggtcatctagg			
*Fgf2*	Fibroblast growth factor 2	(+)ccacacgtcaaactacagctcc	218	60.0	
		(−)gttcgtttcagtgccacatacc			
*Igf1*	Insulin-like growth factor 1	(+)acacaagtagaggaagtgcagg	177	60.0	
		(−)ggaaatgcccatctctgaaatgg			
*Mapt*	Microtubule-associated protein tau	(+)agaggtgaggaagacaggttgg	201	60.0	
		(−)taccttccttctgcccaatacc			
*Ppargc1a*	Peroxisome proliferator-activated receptor gamma, coactivator 1 alpha	(+)cacagagaacagaaacagcagc	236	60.0	
		(−)ctttatgaggaggagtcgtggg			
*Sirt1*	Sirtuin 1	(+)cttgtcctctagttcctgtggc	161	57.2	
		(−)ctccaaatccagatcctccagc			
*Tert*	Telomerase reverse transcriptase	(+)tctagacttgcaggtgaacagc	164	60.0	
		(−)atgctaggttggagatgatgcc			
*CycA*	**Cyclophilin A**	**(+)agcactggggagaaaggatt**	**248**	**58.0**	
		**(−)agccactcagtcttggcagt**			
*Ywhaz*	**Tyrosine 3-monooxygenase/tryptophan, 5-monooxygenase activation protein, z**	**(+)ttgagcagaagacggaaggt**	**136**	**56.1**	
		**(−)gaagcattggggatcaagaa**			
*Tel*	Telomere	(+)ggtttttgagggtgagggtgagggtgagggtgagggt	N/A	54.0	1 cycle 95°C 3 min
		(−)tcccgactatccctatccctatccctatccctatcccta			20 cycles 95°C 15 sec
					20 cycles Tm°C 2 min
					+Melt Curve
*36B4*	**Acidic ribosomal phosphoprotein P0**	**(+)cagcaagtgggaaggtgtaatcc**	**74**	**58.0**	1 cycle 95°C 3 min
		**(−)cccattctatcatcaacgggtacaa**			30 cycles 95°C 15 sec
					30 cycles Tm°C 1 min
					+Melt Curve

Ten nanograms of cDNA with 0.5 μM of each of the forward and reverse primers and 1x SYBR Green FastMix with Rox was used for qRT-PCR using CFX Connect Real-Time PCR Detection System (Bio-Rad, Hercules, CA). A standard curve that was used to determine the PCR efficiency was prepared by serial diluting from 95.24 to 1.5625 ng of cDNA from pooled control samples. One no-template control (NTC)/gene was also subjected to qRT-PCR. The thermocycling conditions for each specific gene can be found in Table 1. Each sample was tested in duplicates, and qRT-PCR for each gene was performed by two separate analysts (IM and RM). Relative target gene expression was determined by normalizing to the two housekeeping genes CycA and Ywhaz (Bonefeld et al., 2008) using the 2^−ΔΔCt^ method as originally described in (Pfaffl, 2001). PCR efficiency was between 90.3 and 108.3%.

#### Telomere length analysis

Genomic DNA was extracted from the ear notch tissue using the Sigma REDExtract N-Amp™ Tissue PCR Kit, according to the manufacturer specifications. Once extracted, DNA quantification and purity analysis was completed using the spectrophotometer NanoDrop 2000 (Thermo Fisher Scientific, Waltham, MA). Genomic DNA from the brain tissue was obtained with the Allprep RNA/DNA Mini kit (Qiagen, Hilden, Germany) as described above. All DNA was diluted to 10 ng/ul for telomere length analysis.

Duplicate PCR reactions using 1 μl of each DNA dilution was carried out in a 20 μl volume using a 1 × SYBR Green FastMix with Rox for qRT-PCR and CFX Connect Real Time PCR Detection System (Bio-Rad, Hercules, CA). Primers for telomeres and the single copy gene 36B4 were designed by the research analyst as previously described by Cawthorn (2002). Primer sequences can be found in Table 1. The final concentrations for primers were Tel forward, 270 nM; Tel reverse, 900 nM; 36B4 forward, 300 nM; and 36B4 reverse, 500 nM. A no-template control was also subjected to qRT-PCR cycling to ensure that the reagents were not contaminated. The thermocycling conditions used for telomere length analysis can also be found in Table 1. The relative telomere to single copy ration (T/S) is used to quantify telomere length. Thus, when T/S = 1, the unknown DNA is identical to the reference DNA, with respect to telomere repeat number and single copy gene number. The T/S ratio was determined to be approximately [2^Ct(telomere)/^2^Ct(36B4)^]^−1^ = −2^−Δ*C*^t. From this analysis, relative telomere length was computed using an equation of linear regression developed by Cawthorn (2002) whereby *y* = 1910.5*x* + 4157, in which *y* = telomere length and *x* = −2^−Δ*C*^t.

#### Statistical analysis

As described above, each experimental condition contained only one male and one female pup from each dam to prevent litter effects. A research analyst blinded to all experimental conditions scored each of the behavioral tests and two research analysts preformed the qRT-PCR for each of the genes analyzed. Three-Way ANOVAs with Injury (mTBI and sham), Diet (HFD, CR, and STD), and Sex (male and female) as factors were run for each of the behavioral and molecular outcomes measured. Pearson's correlation was run for comparison of TL in brain tissue and TL in peripheral skin cells. All analyses were carried out with SPSS 20.0 for Mac, and *p* < 0.05 was considered statistically significant.

## Results

### Behavioral test battery

#### Time-to-right

**Figure 1:**
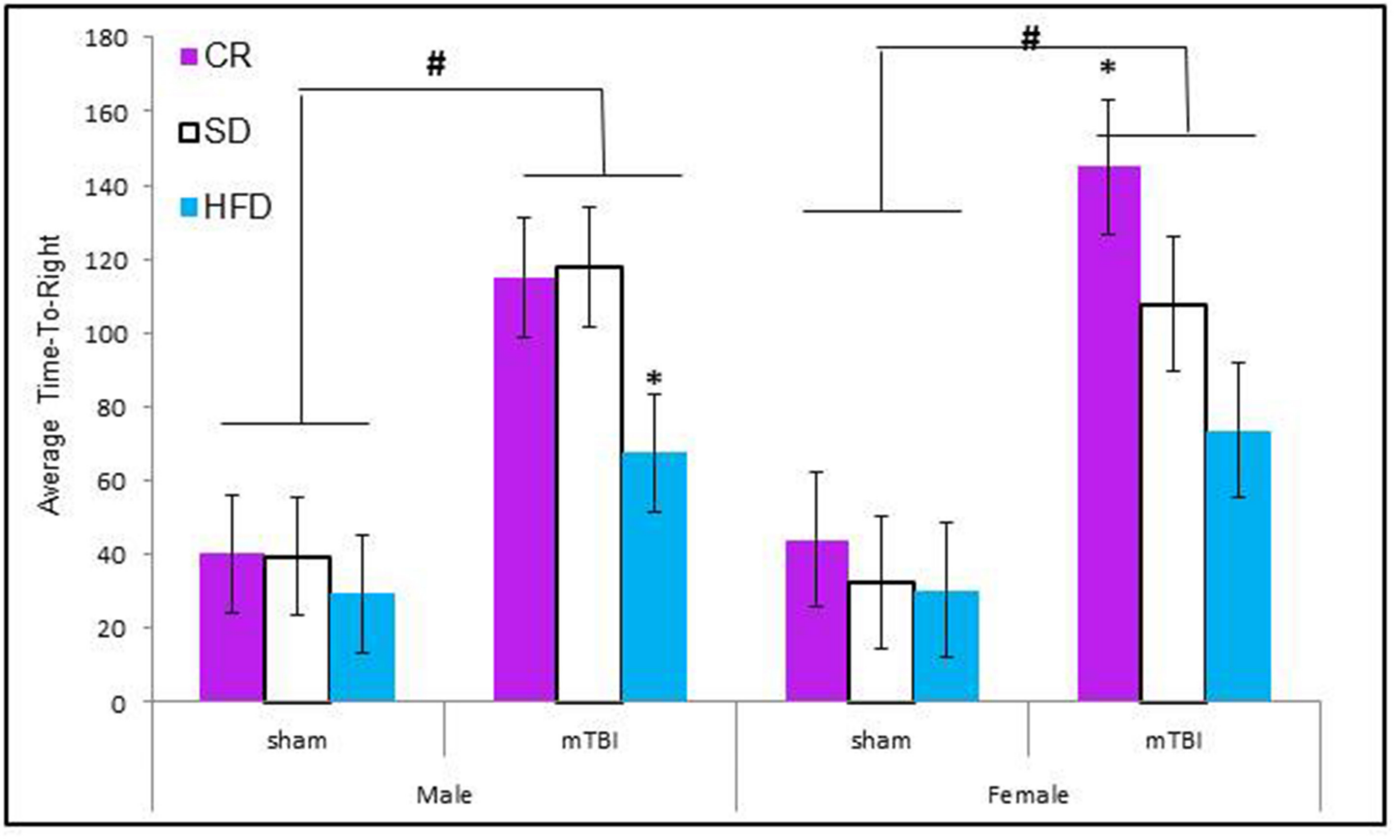
**Graphical representation of the average *time-to-right* following anesthetic and injury induction for rats at P30**. Animals that experienced an mTBI took significantly longer to right themselves and this time was moderated by the animal's diet (#*p* < 0.01; main effect of injury, ^*^p < 0.01; main effect of diet).

Animals from all diet groups with an mTBI exhibited significant increases in the time-to-right following the injury. However, male mTBI-HFD righted significantly faster than mTBI animals in the CR or STD group. The Three-Way ANOVA demonstrated a main effect of injury, *F*_(1, 71)_ = 46.13, *p* < 0.01, and a main effect of diet, *F*_(2, 70)_ = 4.19, *p* = 0.01, but not an effect of sex, *F*_(1, 71)_ = 0.145, *p* = 0.70. None of the interactions were significant, *p*'s > 0.05 see Figure 1.

#### Beam walking foot-slips

**Figure 2:**
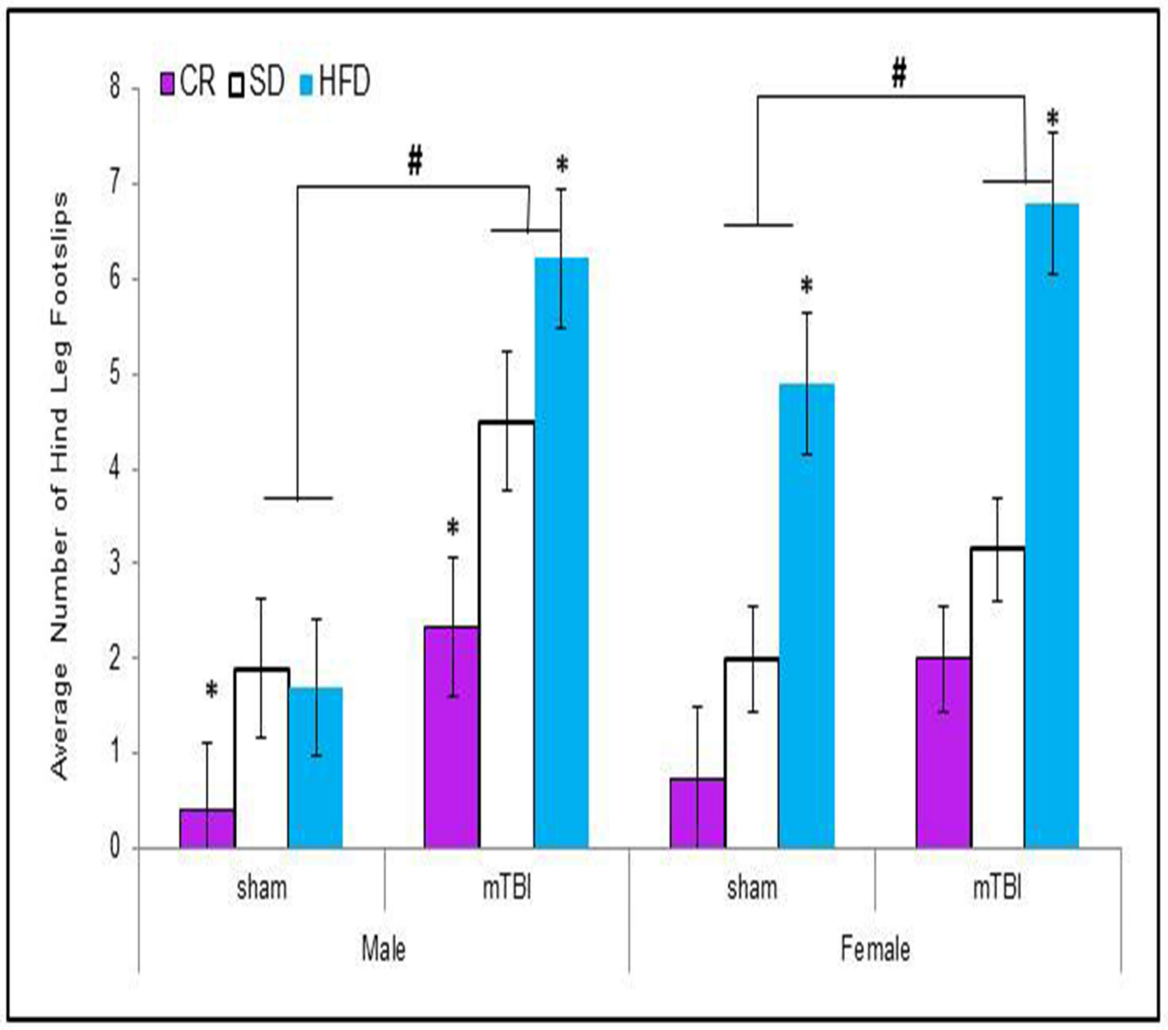
Animals that experienced an mTBI exhibited significantly more hind-leg foot-slips when compared to sham animals on the beam walking task 24 h post-injury; there was also an effect of diet whereby animals on the HFD demonstrated a greater deficit with female-sham animals also performing poorly on the task (#*p* < 0.01; main effect of injury, ^*^p < 0.01; main effect of diet).

Animals that experienced a mTBI at P30 exhibited significantly more hind-leg foot-slips on the beam walking task than control animals. This was more prevalent in HFD animals. The Three-Way ANOVA demonstrated a main effect of injury, *F*_(1, 71)_ = 14.11, *p* < 0.01, and a main effect of diet, *F*_(2, 70)_ = 12.36, *p* < 0.01, but not of sex, *F*_(1, 71)_ = 0.47, *p* = 0.49. None of the interactions were significant see Figure 2.

#### Open field

**Figure 3:**
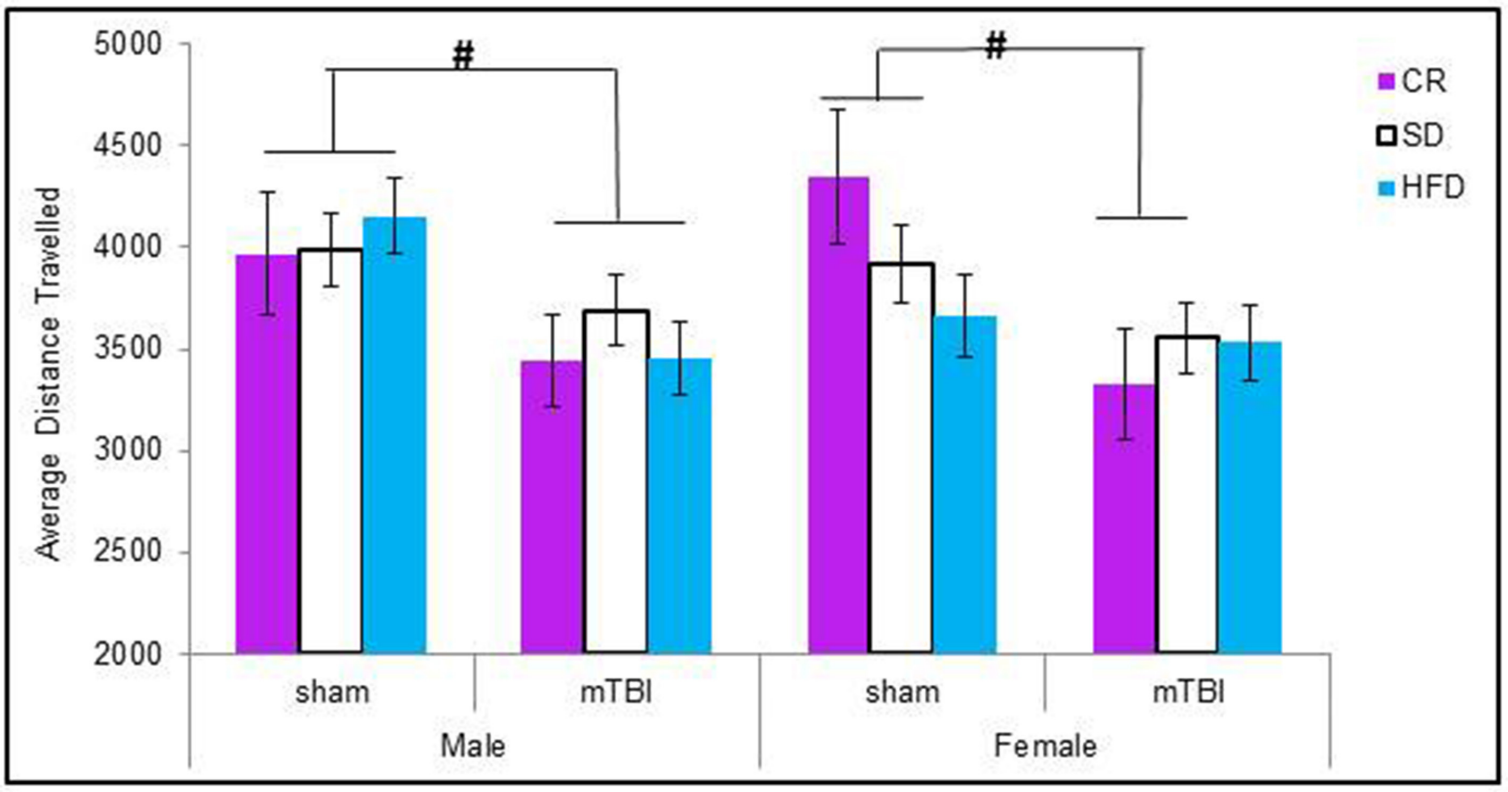
**On postnatal day 32, animals that experienced a mTBI spent significantly less time exploring the openfield enclosure as exhibited by a decrease in the average distance covered**. Diet did not affect the distance animals traveled (#*p* < 0.05; main effect of injury).

When examining the total distance traveled, male animals in all of the dietary groups displayed a reduction in locomotion following the mTBI, whereas only females in the CR group exhibited the same reduction after the injury. The Three-Way ANOVA only demonstrated a main effect of injury, *F*_(1, 71)_ = 15.17, *p* < 0.01. None of the other factors were significant and there were no significant interactions, *p*'s > 0.05 see Figure 3.

#### Elevated plus maze

**Figure 4:**
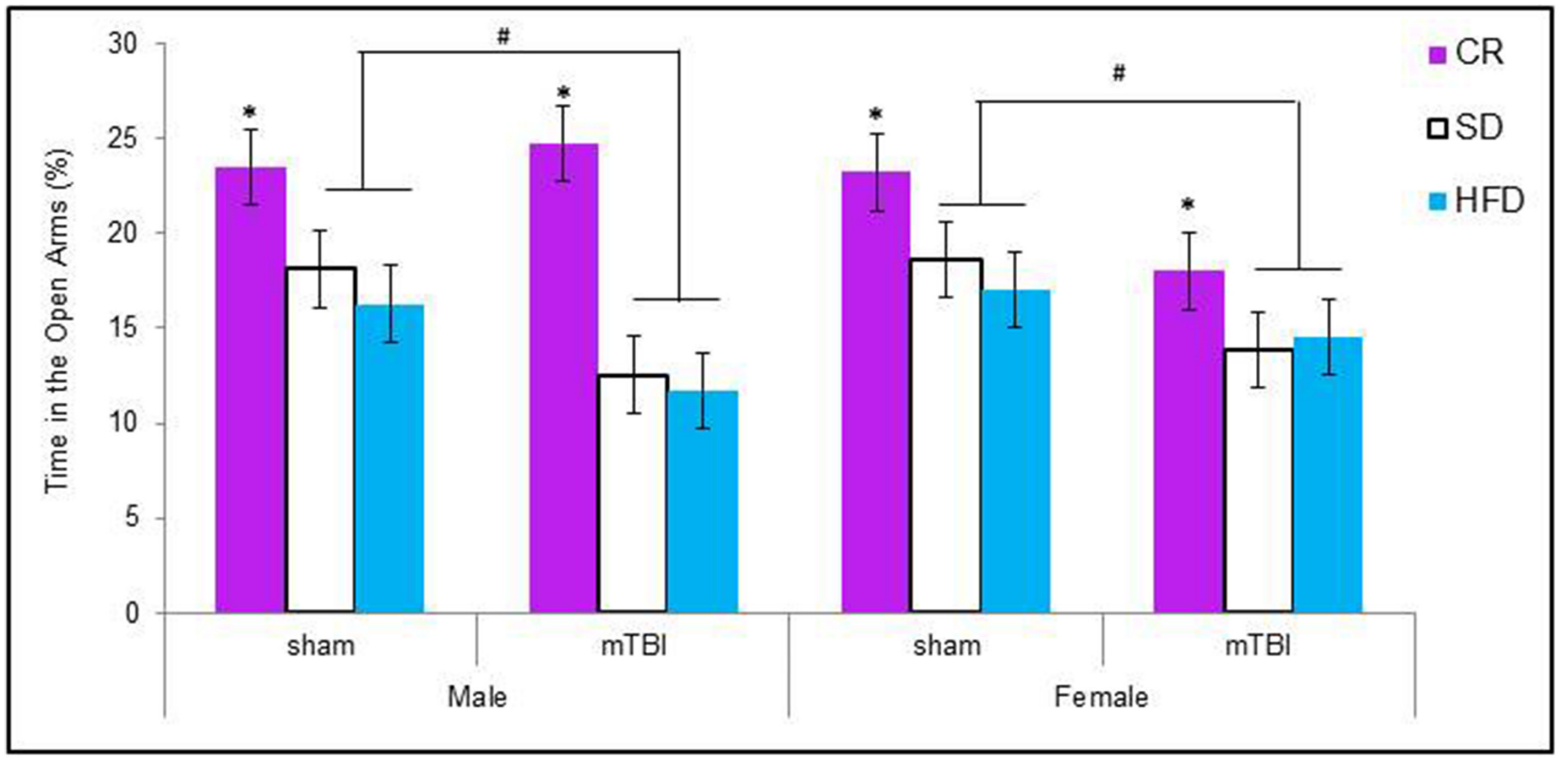
**Graphical representation of the average amount of times male and female animals with a mTBI or sham injury spent in the open arms of the elevated plus maze**. Both male and female animals on the HFD and SD spent significantly less time in the open arms following the brain injury. The amount of time in the open arms was not altered for CR animals with a mTBI (#*p* < 0.01; main effect of injury, ^*^p < 0.01; main effect of diet).

Male animals in the HFD and STD groups that received a P30 mTBI spent less time in the open arms of the EPM. Conversely, female animals in the CR and STD groups that received a P30 mTBI spent less time in the open arms of the EPM. The Three-Way ANOVA demonstrated a main effect of injury, *F*_(1, 71)_ = 6.75, *p* = 0.01, and a main effect of diet, *F*_(2, 70)_ = 8.63, *p* < 0.01, but not of sex, *F*_(1, 71)_ = 0.03, *p* = 0.86. None of the interactions were significant, *p*'s > 0.05 see Figure 4.

#### Novel context mismatch

**Figure 5:**
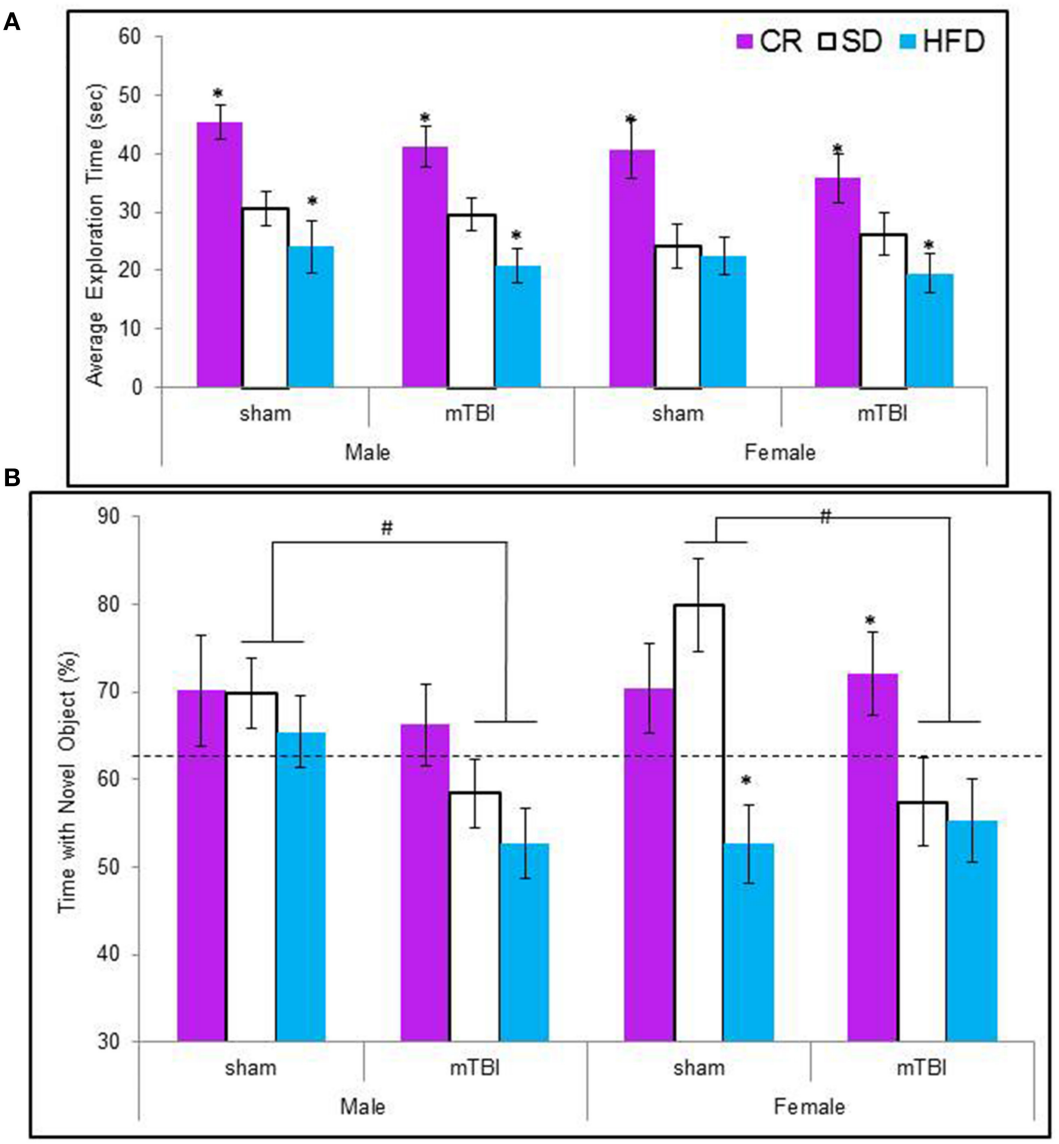
**(A)** Average amount of time animals in each experimental condition spent exploring the objects during the 5 min novel context mismatch probe trial. All animals in the CR group spent a greater amount of time investigating the objects whereas animals with an early mTBI in the HFD groups spent the least amount of time investigating the objects (^*^p < 0.01; main effect of diet). **(B)** Illustrative representation of the time animals spent with the novel object during the probe trial. Both male and female animals with an mTBI in the STD and HFD groups were unable to distinguish the novel object and spent equal time with the new and old objects. CR animals were able to complete the task without impairment following the mTBI (#*p* < 0.01; main effect of injury, ^*^p < 0.01; main effect of diet).

Animals maintained on the CR diet spent significantly more time exploring the objects in the test context, see Figure 5A The CR animals also spent a greater percentage of their time with the novel object regardless of their injury classification. Male and female animals with an mTBI from the STD and HFD groups exhibited a deficit on this task by spending significantly less of their time with the novel object. Female-sham animals–HFD also exhibited deficits on the NCM task, see Figure 5B. The Three-Way ANOVA for total exploration time demonstrated a main effect of diet, *F*_(2, 70)_ = 25.60, *p* < 0.01, but not of sex, *F*_(1, 71)_ = 3.38, *p* = 0.07, or of injury, *F*_(1, 71)_ = 1.27, *p* = 0.26. None of the interactions were significant, *p*'s > 0.05. The Three-Way ANOVA for time spent with the novel object demonstrated a main effect of injury, *F*_(1, 71)_ = 5.37, *p* = 0.02, a main effect of diet, *F*_(2, 70)_ = 7.19, *p* < 0.0, but no effect of sex, *F*_(1, 71)_ = 0.01, *p* = 0.96. The interactions were not significant.

#### Morris water task

**Figure 6:**
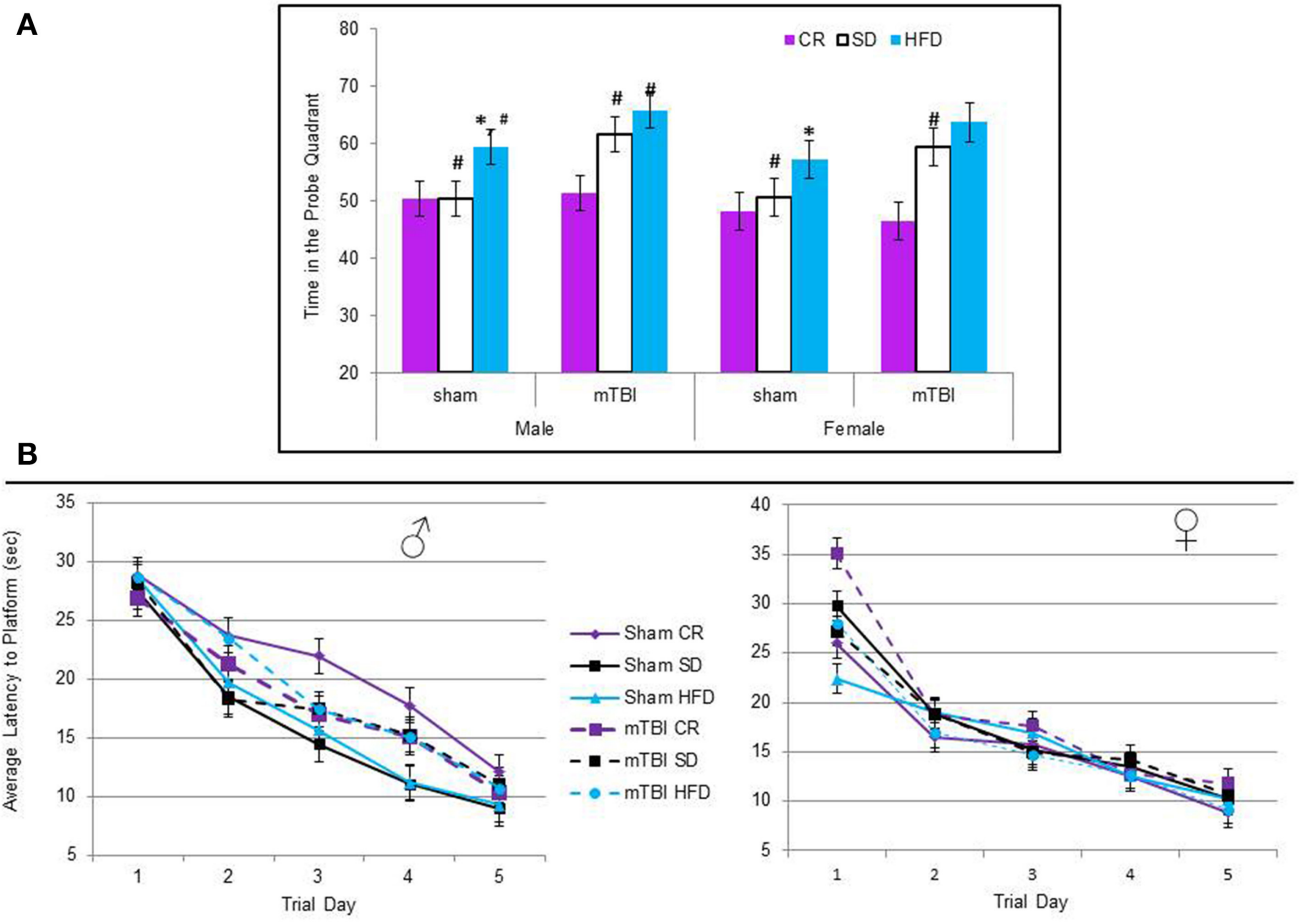
**(A)** Average percentage of time animals spent in the quadrant that had previously contained the hidden platform on the probe trial of the Morris water task. The protocol employed in this study utilized a 60 s probe trial; under these conditions, normal animals will typically spend the first 50% of their time searching in the area that previously contained the platform, but upon discovery that it is not there, switch strategies and search for the platform in other regions for the remaining 50% of the time. Animals on the HFD and STD spent significantly more time than would be expected in the platform quadrant if they had experienced an mTBI. In addition, male sham animals on the HFD also spent more time in the platform quadrant than the CR and STD sham animals (^*^p < 0.01; main effect of injury, #*p* < 0.01; main effect of diet). **(B)** There was no significant difference in the time it took animals to learn the location of the hidden platform over the 5 trial days, regardless of injury status or dietary intake (*p* > 0.05).

All of the animals were able to learn the location of the hidden platform in similar time-frames, indicating no deficits in spatial memory were induced by diet or the injury. The repeated measures ANOVA with trial day, sex, injury, and diet as factors failed to demonstrate a specific main effect when sphericity was assumed, *F*_(2, 60)_ = 0.67, *p* = 0.68 see Figure 6B. However, on the probe trial, when the platform was removed, animals fed the STD or HFD with a mTBI spent significantly more time in the probe quadrant. This was also true for male HFD animals without a mTBI. The Three-Way ANOVA demonstrated a main effect of injury, *F*_(1, 71)_ = 5.09, *p* = 0.02, and of diet, *F*_(2, 70)_ = 8.52, *p* < 0.01, but not of sex, *F*_(1, 71)_ = 1.19, *p* = 0.28. None of the interactions were significant, see Figure 6A.

### Molecular and telomere length analysis

**Figure 7:**
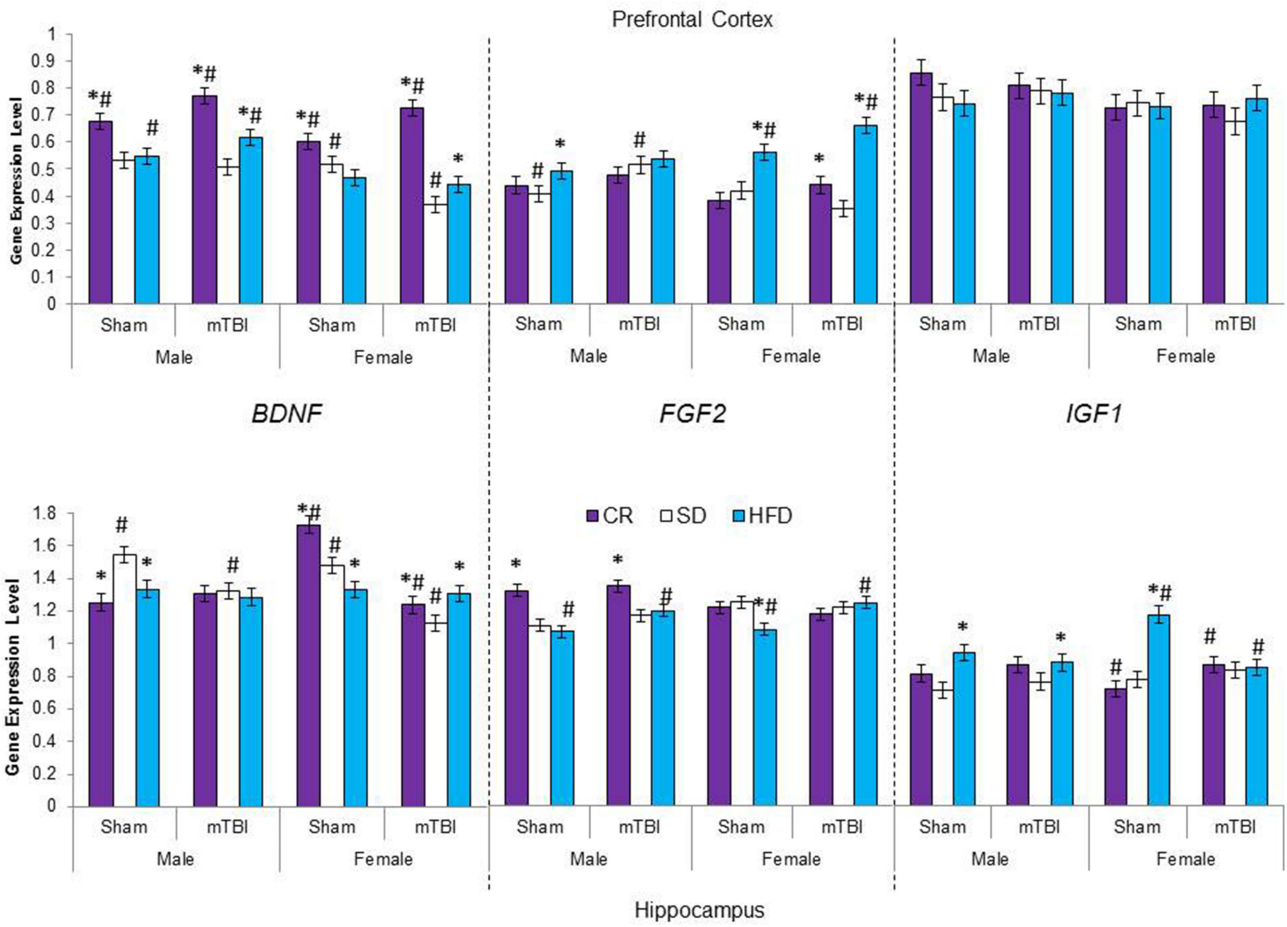
**Average change in gene expression for male and female rats at the time of sacrifice (~P47) for the 3 growth factor genes (*BDNF, FGF2, IGF1*) in the two different brain regions examined**. Significant main effects of diet (^*^p < 0.05) and significant main effects of injury (#*p* < 0.05) are indicated on the graph; all comparisons are made between the experimental group and the control group.

**Figure 8:**
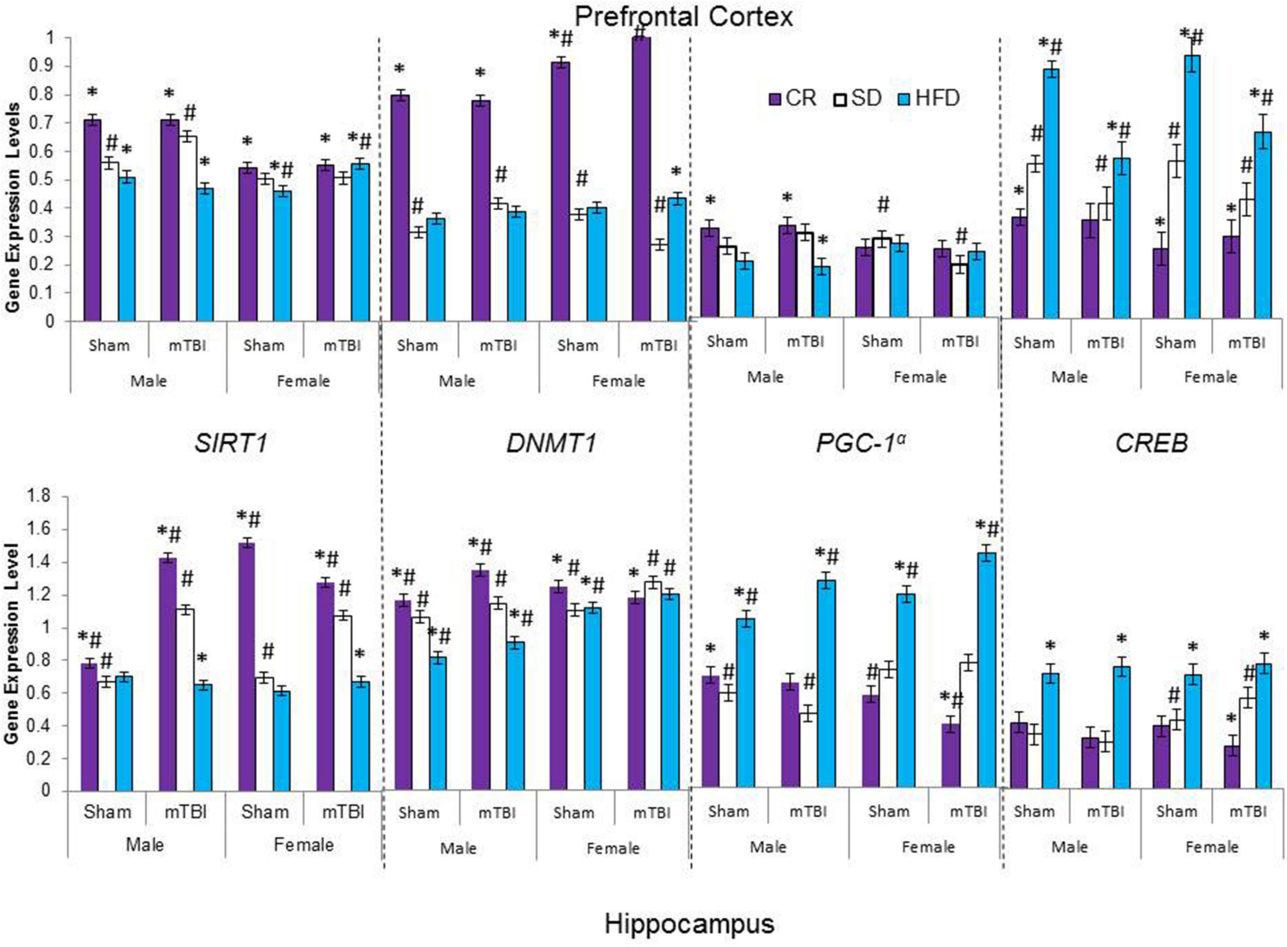
**Average change in gene expression for male and female rats at the time of sacrifice (~P47) for the 4 epigenetic regulator genes (*SIRT1, DNMT1, CREB, PGC-1α*) in the two different brain regions examined**. Significant main effects of diet (^*^p < 0.05) and significant main effects of injury (#*p* < 0.05) are indicated on the graph; all comparisons are made between the experimental group and the control group. A significant interaction was present in expression of *DNMT1* in the PFC of female animals whereby the mTBI decreased expression in animals on the SD, but increased expression for animals on CR.

**Figure 9:**
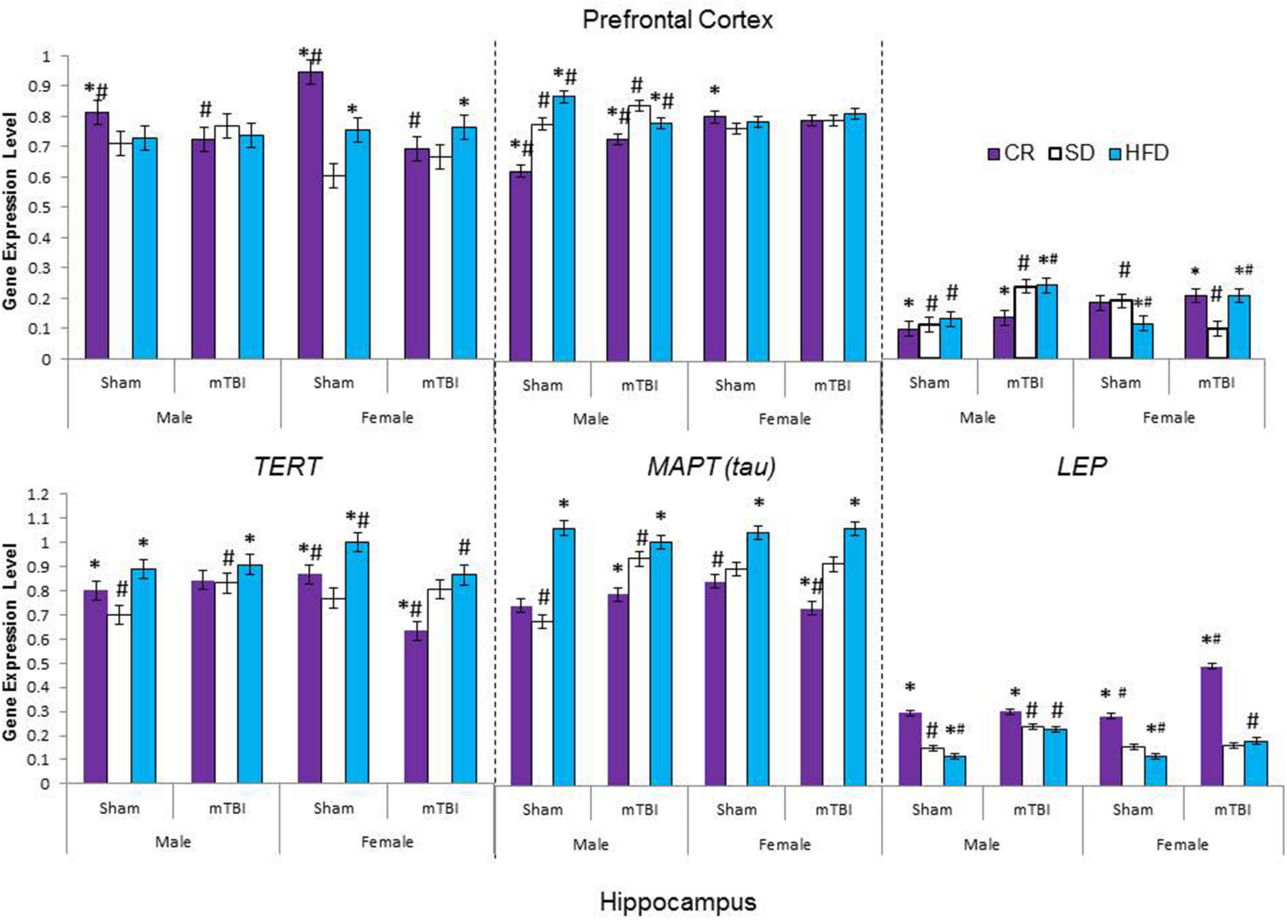
**Average change in gene expression for male and female rats at the time of sacrifice (~P47) for the 3 neurological markers (*TERT, Tau, LEP*) in the two different brain regions examined**. Significant main effects of diet (^*^p < 0.05) and significant main effects of injury (#*p* < 0.05) are indicated on the graph; all comparisons are made between the experimental group and the control group. A significant interaction existed for males in the PFC whereby levels of *MAPT (tau)* were increased in animals on the SD and CR following the mTBI, but decreased following the mTBI in animals fed the HFD.

**Figure 10:**
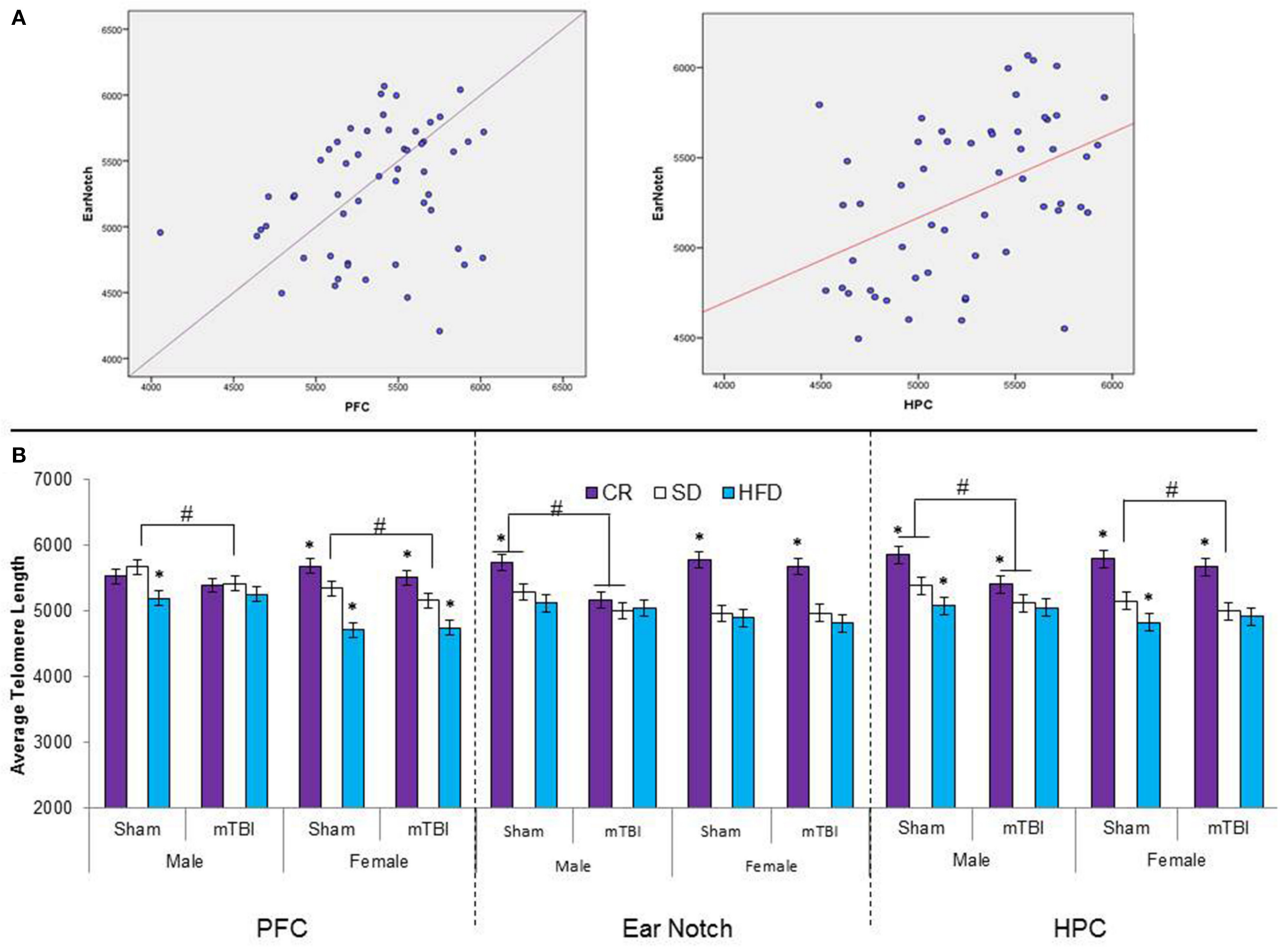
**(A)** Upper portion of the figure contains the scatterplots for the correlational analysis between TL in the PFC and peripheral skin cells from ear notch samples along with a correlational analysis between TL in the HPC and skin cells from the ear notch samples. **(B)** Illustrates average telomere length for skin cells (ear notch samples), and tissue from the PFC and HPC for male and female rats that had experienced early mTBI or sham injuries that were fed a HFD, CR, or SD (^*^p < 0.01; main effect of injury, #*p* < 0.01; main effect of diet). A significant three-way interaction was found in the HPC whereby both experimental diets affected the average TL in sham animals, with the mTBI resulting in decreased TL for male animals on CR and SD animals, and only SD-female animals.

The large amount of data created from Three-Way ANOVAs for 10 genes of interest from two distinct brain regions and three separate analysis of telomere length has been summarized in Tables 2–4. All analyses were carried out using *n* = 5 for each experimental condition, i.e., 5 HFD mTBI ♀s. Graphical representations of the data can be found in Figures 7–10. Briefly however, correlational analysis between TL in the brain regions and TL in peripheral skin cells at the time of sacrifice demonstrates significant correlation between TL skin + TL PFC with *r* = 0.497, *p* < 0.001, in addition to a *r* = 0.30, *p* < 0.02 for TL skin + TL HPC. Diet had the largest effect on gene expression in both brain regions examined; changes in gene expression differ with respect to the brain region of interest; and peripheral TL reflects TL in the PFC and HPC and may therefore serve as a reliable peripheral marker of brain changes.

**Table 2:**
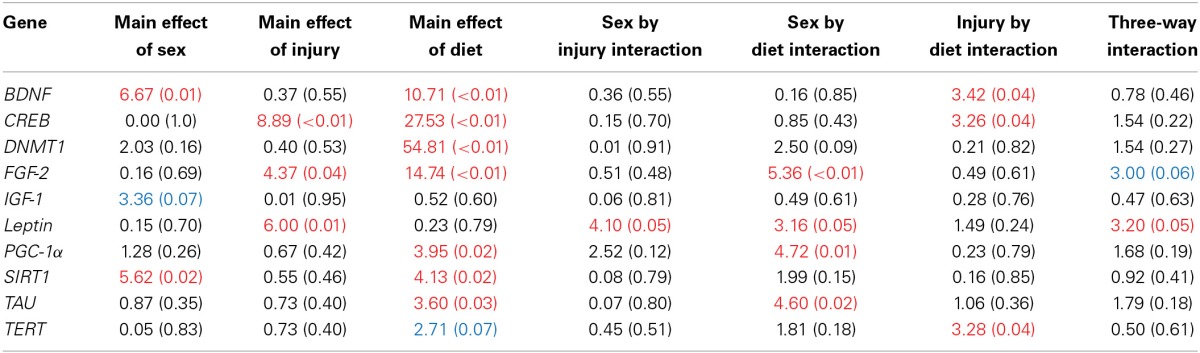
**Summary of the Three-Way ANOVA results for gene expression changes in the PFC for rats fed the HFD or CR, following mTBI or sham injury at the time of sacrifice (*n* = 60) [*F* value (*p*)]**.

Red values are significant effects at p < 0.05, whereas blue values were trends toward significant effects with p < 0.07.

**Table 3:**
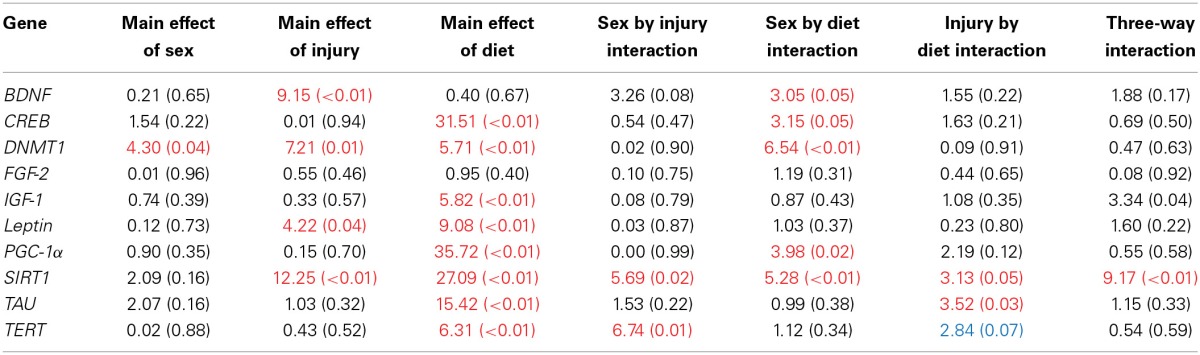
**Summary of the Three-Way ANOVA results for gene expression changes in the HPC for rats fed the HFD or CR, following mTBI or sham injury at the time of sacrifice (*n* = 60) [*F* value (*p*)]**.

Red values are significant effects at p < 0.05, whereas blue values were trends toward significant effects with p < 0.07.

**Table 4:**
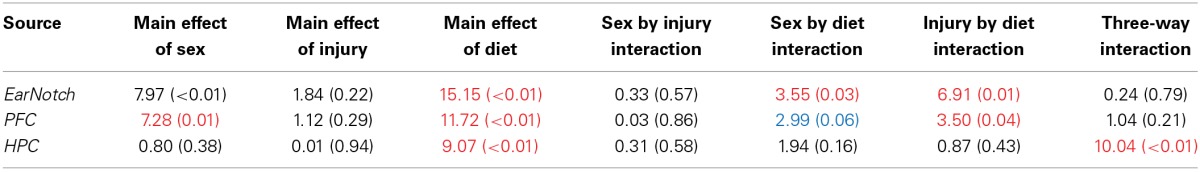
**Summary of Three-Way ANOVA results for telomere length analysis in peripheral skin cells (ear notch), along with tissue from the HPC and PFC, for male and female rats fed the HFD, STD, or CR at the time of sacrifice (~P45)**.

Red values are significant effects at p < 0.05, whereas blue values were trends toward significant effects with p < 0.07.

## Discussion

Considering the great deal of inter-individual variation that exists, the likelihood of any given individual developing a complex disorder like post-concussion syndrome, is a joint function of cumulative exposure to risk factors and the individual's predisposition (Entringer et al., 2012). Owing to the fact that individual vulnerability or resiliency is based upon genetic and epigenetic patterning, which is also influenced by environmental circumstances (Jirtle and Skinner, 2007), environmental experiences may play a greater role than predisposition. This study examined multiple risk factors that could contribute to susceptibility or resiliency to post-concussive symptoms and clearly demonstrated that dietary intake altered outcomes and affected the molecular changes induced by and compensating for the mTBI. Rats that were maintained on the HFD displayed poor recovery when compared to the STD animals and those on CR. In some circumstances animals on the CR diet appeared to be resilient to the injury and displayed behavioral characteristics similar to sham animals. Dietary intake may therefore contribute to differential susceptibility to post-concussion syndrome risk and resiliency. The results from the TL analysis demonstrate that the use of TL as a peripheral marker for neurological function in the context of mTBI may provide researchers and clinicians with a useful tool for the identification of individuals at risk for prolonged symptomology and recovery.

In addition, this study was able to demonstrate a relationship between dietary consumption and specific genetic and epigenetic changes that may help explain differential responses to the same brain injury. Of importance, this study was consistent with many other studies in our laboratory and the laboratories of others, that have demonstrated significant sex-differences in behavioral outcomes and changes in gene expression following mTBI (e.g., Max et al., 1998; Mychasiuk et al., 2014a,b, 2015; Bay et al., 2009; Bazarian et al., 2010). Sex-effects were found in the prefrontal cortex (4/10 genes) and extensively in the hippocampus (8/10 genes), with a majority of these genes exhibiting sex by diet interactions. Interestingly, on a majority of the behavioral tests examined, we failed to show sex-differences despite significant effects of diet and injury. It appears that although, male and female animals end up with similar performances on behavioral tasks when exposed to similar injuries and diets, they often reach this endpoint by utilizing different epigenetic strategies. This may be a result of pre-existing sex-differences in epigenetic regulators such as methyl transferases (McCarthy et al., 2009), or related to differential coping strategies as research has shown that the effects of maternal diet during the prenatal period are inherited distinctly to offspring depending on their sex (Dunn et al., 2011). It is also possible that varied sex-dependent rates of cortical brain maturation (Kolb and Whishaw, 2008) and pre-existing differences in sex hormones and neurotrophic factors (Molteni et al., 2001; Wu et al., 2009) are responsible for the substantial variation in epigenetic response to the dietary manipulation and mTBI. This is an important finding as it may imply that males and females will require different treatment strategies regardless of similar symptomology presentation.

### Experience dependent changes to telomere length (TL)

When TL drops below a certain threshold, the cell has limited capacity to respond to stress, reduced proliferative potential, and may actually enter programmed cell death (Blasco, 2005). TL is a homeostatic relationship dependent upon; (a) an individual's initial telomere length, as generated during fetal development, (b) telomere attrition, as determined by rates of cellular replication, and cumulative exposure to DNA damaging agents, and (c) the offsetting effects of telomerase, based upon individual differences in telomerase efficiency (Eisenberg, 2011; Liu et al., 2004). Therefore processes that alter initial TL likely confer significant susceptibility to later life pathologies (Entringer et al., 2012). In this study, both peripheral markers and actual brain tissue, chronic exposure to the dietary manipulations significantly altered TL early in life. In female rats, exposure to the HFD significantly reduced TL in peripheral cells, the prefrontal cortex and the HPC, whereas males maintained on this diet exhibited significant reductions in TL in brain tissue (PFC and HPC), but not ear notch samples. Conversely, similar to other studies (Vera et al., 2013) chronic CR exposure increased TL in peripheral cells of both males and females. What's more, rats on the HFD with shortened PFC telomeres at the time of injury were also more likely to experience negative behavioral symptomology following the mTBI. In conjunction with other research (Valdes et al., 2005; Cassidy et al., 2010; Paul, 2011), these findings provide support that HFDs are capable of affecting the innate biology of individuals, which in turn may increase susceptibility to further pathologies. In addition, in brain tissue after the mTBI, male rats on the SD that had an mTBI had shorter TLs in the PFC and HPC and female rats on a similar diet exhibited reductions in TL in the PFC following the mTBI. These findings suggest that early brain injuries, even those classified as mild, have the potential to alter telomere length and increase an individual's risk for neurodegeneration in the long-term. Of significant importance, although not identical, the TL patterns obtained from peripheral skin cells (ear notches) were very similar to those obtained from the brain tissue. Although it will require further investigation with a greater sample size, TL from peripheral cells may be a useful biomarker for the prediction of susceptibility and resiliency to poor outcomes following mTBI.

### Influence of diet and injury on molecular patterns

Although the exact mechanisms underlying the neurological effects of CR and HFDs have not been differentiated, there are certain epigenetic pathways known to be intricately involved. For example, the benefits of CR have been linked to up-regulation of *DNMT*s, *SIRT1*, *TERT*, *PGC-1α*, and *BDNF* (Li et al., 2011). On the contrary, HFDs have been associated with altered *BDNF* and *CREB* expression, up-regulation of *TAU*, and *LEP* (leptin) along with *IGF1* insensitivities (Wu et al., 2003; Gomez-Pinilla, 2008; Pistell et al., 2010). It is clear from the literature that a single gene or genetic marker in unlikely to fully explain resiliency or susceptibility to poor outcomes following an mTBI. Thus, this study examined key factors that could be used to further understanding of the molecular networks involved in response to mTBI under normal conditions and following dietary manipulation.

Caloric restriction is believed to increase lifespan and delay the onset of age-related pathologies in part by influencing epigenetic processes via DNA methylation and histone modification (Masoro, 2005; Li et al., 2011). Modifications to these epigenetic processes are thought to increase synaptic plasticity, maintain genome stability, regulate cellular stress response pathways, decrease inflammation, and control energy expenditure and metabolism (Masoro, 2005; Lavu et al., 2008; Li et al., 2011). Prior literature indicates that CR activates *DNMT1* and *SIRT1* which in turn can lead to up-regulation of *TERT* and *PGC-1α*; a process important for maintaining chromatin stability and reversing aberrant gene expression changes when the cell is responding to stress. *PGC-1α* is a transcriptional co-activator that regulates genes involved in energy metabolism and is a direct link between external stimuli and the regulation of mitochondrial biogenesis (Lavu et al., 2008). *TERT* promotes cellular survival under conditions of cellular stress by participating in chromosomal repair, decreasing oxidative stress and maintaining telomeres. In line with previous literature, animals in this study maintained on CR demonstrated increased gene expression in all four of these genes in both brain regions examined (except *PGC-1α* in females). Animals on CR that had experienced an early mTBI also exhibited an overall increase in expression of these four genes, as would be expected from the literature. However the profiles were different from those of CR-Sham animals, possibly exhibiting a difference in genetic response that results from the interaction of the CR and the mTBI induced stress.

*SIRT1* is also believed to work in concert with *CREB*, whereby *CREB* expression activates *SIRT1*. Research has demonstrated that *CREB* is metabolically regulated in the neocortex and HPC and activation of *CREB* is required for the neuronal plasticity, stress resistance and cellular survival identified following CR (Fusco et al., 2012). Contrary to expectation, all of the animals maintained on CR in this study exhibit a down-regulation or lower levels of *CREB*. As tissue was sampled 17 days after the mTBI and *CREB* is also down-regulated in STD–mTBI animals, it is possible that there was an initial rise in *CREB* immediately after the injury that initiated the *SIRT1* signaling cascade to cope with the cellular stress, but has since been inhibited as part of a negative-feedback loop. The overall modification to these specific genes (*CREB, DNMT1, PGC-1α, SIRT1*, and *TERT*), may have provided animals in this group with additional compensatory mechanisms to overcome the cellular stress induced by the mTBI and thereby facilitating normal behavioral function. In addition, CR is also believed to increase levels of *BDNF* and this is thought to contribute to the increased plasticity and neuroprotection identified in calorie restricted populations (Cheng et al., 1997; Lee et al., 2002). Consistent with this, *BDNF* levels were significantly higher in the PFC of CR animals with or without an early mTBI, but significantly lower in the PFC of female animals maintained on the HFD.

Diets high in fat and sugar decrease levels of *BDNF* to the extent that it compromises neuroplasticity and cognitive function, in addition to aggravating outcomes associated with neurological insult and possibly contributing to PTSD (Wu et al., 2004; Kaplan et al., 2010). A decrease in *BDNF* expression was found in both brain regions following mTBI in animals fed the STD, and likely contributed to the poor behavioral outcomes and lingering symptomology identified. Interestingly, this abnormal reduction in *BDNF* levels was also more prevalent in animals fed the HFD, who also demonstrated poor performances on the behavioral tasks. The effect of *BDNF* on metabolism and synaptic plasticity appears to involve *IGF1* and *FGF2*. At normal concentrations, *IGF1* supports nerve growth and differentiation, neurotransmitter synthesis and release, as well as synaptic plasticity, however at substantially higher levels *IGF1* is positively correlated with many cancers, diabetes and neurodegeneration (Rabinovsky, 2004). What's more, recent research has indicated that neurodegenerative and demyelinating pathologies may be linked to abnormal elevations in *FGF2* (Tatebayashi et al., 2003; Butt and Dinsdale, 2005). *IGF1* was significantly increased in the HPC of females on the HFD with an mTBI, and in the HPC of all males on the HFD, whereas increases in *FGF2* expression were demonstrated in the PFC of most animals that had experienced an early mTBI. These epigenetic modifications in conjunction with the increased levels of *MAPT (TAU)* identified in animals with an mTBI or on the HFD provide multiple pathways that may lead to neurodegenerative symptomologies.

Finally, *LEP* facilitates synaptic plasticity in the hippocampus, reduces levels of stress hormones, and acts as a neurotrophic factor during brain development (Barzilai and Gupta, 1999; Harris, 2000; Ferezou-Viala et al., 2007). Under normal conditions, as levels of *LEP* rise, an individual's motivation to eat is reduced (Harris, 2000). Interestingly, in this study, *LEP* levels were generally decreased in sham animals on the HFD and increased in CR sham animals; indicating that animals on the HFD would have a heightened motivation to eat, whereas CR animals would experience reduced motivation to eat. In addition, because *LEP* acts as a neurotrophic factor that increases plasticity and reduces stress hormones, the elevated levels identified in CR animals may have provided them with an additional mechanism to improve neuroplasticity and compensatory responses following the mTBI that would not have been available to animals on the HFD.

### Dietary modification of behavioral outcomes following mTBI

Previous studies in our laboratory have used the behavioral test battery described here as a measure of experimental post-concussion syndrome (Mychasiuk et al., 2014a). Based on the current results examining balance and motor control, short-term working memory and an executive function task involving perseveration, animals maintained on CR appear to be resilient to the mTBI and exhibited behaviors that were indistinguishable from CR sham animals. While other studies have demonstrated that CR following moderate-to-severe TBI improves performance on spatial memory tasks (Davis et al., 2008; Rich et al., 2010), this study is the first to show that long-term exposure to a reduced calorie diet may actually prevent or reduce susceptibility to mild neurological insults.

In contrast, and consistent with our hypothesis, rats maintained on the HFD demonstrated greater susceptibility to poor outcomes after the mTBI and even demonstrated poorer performance in the absence of the mTBI. These results are similar to those found by the laboratory of Gomez-Pinilla (Molteni et al., 2002; Wu et al., 2004), who demonstrated chronic exposure to a HFD in and of itself, reduced synaptic plasticity and learning in rats. Although the HFD only exacerbated mTBI effects in two of the behavioral paradigms (beam walking and NCM exploration time), in the other tests the HFD animals demonstrated behavioral impairments at least equal to the SD animals. These finding suggest an increased susceptibility to post-concussion syndrome. In comparison, other studies examining the effects of HFD on ischemic stroke and moderate TBI have demonstrated that HFDs worsen a broader range of outcomes (Wu et al., 2003; Langdon et al., 2011). There are two possible reasons that we did not see this widespread effect. First, it is possible that the mTBI/concussion was mild enough that the young brain was able to compensate for the injury without further exacerbation from the HFD. Second, and more likely, the time period that animals were maintained on the diet was too short, had the injury and examination of behavioral symptoms occurred later in life, it is possible the HFD would have prevented normal recovery from the mTBI. Longer exposure to a HFD may impose greater risk to the brain, making it more likely that negative consequences would result if the injury was incurred at a later age.

In summary, the behavioral outcomes and gene expression changes identified in this study demonstrate that dietary manipulations differentially alter baseline characteristics and therefore contribute to predisposing heterogeneity. This is important for the study of pediatric concussion because genetic patterns and behavioral abilities will differ significantly before the actual mTBI. Therefore, even in situations where injuries appear to be similar, daily and past experiences such as an individual's dietary intake will dramatically influence outcomes. Owing to the fact that a plethora of genes and pathways are involved in the propagation of neuroprotection or neurodegeneration following even mild injuries, treatment strategies may be more proficient if they capitalize on indiscriminate techniques like exercise, rather than single molecule “magic bullets.” In addition, underlying differences in TL prior to and following the mTBI have to potential to not only alter the long-term trajectories of these individuals, but also act as predictive biomarkers for risk and resiliency. Taken together, the findings from this study provide molecular and genetic support to the hypothesis that dietary intake may confer differential susceptibility to poor outcomes following mTBI.

### Conflict of interest statement

Dr. Michael Esser reports funding from Alberta Children's Hospital Research Institute (ACHRI) and the Alberta Children's Hospital Foundation. Dr. Richelle Mychasiuk reports funding from ARCHI. Irene Ma has no financial interests to disclose. Harleeen Hehar reports funding from the Markin USRP for Health and Wellness.

## References

[B1] Barlow K. Crawford S. Stevenson A. Sandhu S. Belanger F. Dewey D. (2010). Epidemiology of postconcussion syndrome in pediatric mild traumatic brain injury. Pediatrics 126, e374–e381. 10.1542/peds.2009-0925 20660554

[B2] Barzilai N. Gupta G. (1999). Revisiting the role of fat mass in the life extension induced by calorie restriction. J. Gerontol. A Biol. Sci. Med. Sci. 54, B89–B96. 10.1093/gerona/54.3.B89 10191831

[B3] Bay E. Sikorskii A. Saint-Arnault D. (2009). Sex-differences in depressive symptoms and their correlates after mild-to-moderate traumatic brain injury. J. Neurosci. Nurs. 41, 298–309. 10.1097/JNN.0b013e3181b6be81 19998681

[B4] Bazarian J. Blyth B. Mookerjee S. He H. McDermott M. (2010). Sex differences in outcomes after mild traumatic brain injury. J. Neurotrauma 27, 527–539. 10.1089/neu.2009.1068 19938945 PMC2867588

[B5] Blasco M. (2005). Telomeres and human disease: ageing, cancer, and beyond. Nat. Rev. Genet. 6, 611–622. 10.1038/nrg1656 16136653

[B6] Bonefeld B. Elfving B. Wegener G. (2008). Reference genes for normalization: a study of rat brain tissue. Synapse 62, 302–309. 10.1002/syn.20496 18241047

[B7] Brown C. Rhee P. Neville A. Sangthong B. Salim A. Demetriades D.. (2006). Obesity and traumatic brain injury. J. Trauma 61, 572–576. 10.1097/01.ta.0000200842.19740.38 16966989

[B8] Butt A. Dinsdale J. (2005). Fibroblast growth factor 2 mediated disruption of myelin-forming oligodendrocytes *in vivo* is associated with increased tau immunoreactivity. Neurosci. Lett. 375, 28–32. 10.1016/j.neulet.2004.10.060 15664117

[B9] Cassidy A. De Vivo I. Liu Y. Han J. Prescott J. Hunter D.. (2010). Associations between diet, lifestyle factors, and telomere length in women. Am. J. Clin. Nutr. 91, 1273–1280. 10.3945/ajcn.2009.28947 20219960 PMC2854902

[B10] Cawthorn R. (2002). Telomere measurement by quantitative PCR. Nucleic Acids Res. 30:e47. 10.1093/nar/30.10.e47 12000852 PMC115301

[B11] Cheng Y. Gidday J. Yan Q. Shah A. Holtzman D. (1997). Marked age-dependent neuroprotection by brain-derived neurotrophic factor against neonatal hypoxic-ischemic brain injury. Ann. Neurol. 41, 521–529. 10.1002/ana.410410416 9124810

[B12] Davis L. M. Pauly J. Readnower R. Rho J. Sullivan P. (2008). G. Fasting is neuroprotective following traumatic brain injury. J. Neurosci. Res. 86, 1812–1822. 10.1002/jnr.21628 18241053

[B13] DeWitt D. Perez-Polo R. Hulsebosch C. Dash P. Robertson C. (2013). Challenges in the development of rodent models of mild traumatic brain injury. J. Neurotrauma 30, 688–701. 10.1089/neu.2012.2349 23286417

[B14] Dunn G. Morgan C. P. Bale T. (2011). Sex-specificity in transgenerational epigenetic programming. Horm. Behav. 59, 290–295. 10.1016/j.yhbeh.2010.05.004 20483359

[B15] Eckless-Smith K. Clayton D. Bickford P. Browning M. (2000). Caloric restriction prevents age-related deficits in LTP and in NWDA receptor expression. Mol. Brain Res. 78, 154–162. 10.1016/S0169-328X(00)00088-7 10891595

[B16] Eisenberg D. (2011). An evolutionary review of human telomere biology: the thrifty telomere hypothesis and notes on potential adaptive paternal effects. Am. J. Hum. Biol. 23, 149–167. 10.1002/ajhb.21127 21319244

[B17] Entringer S. Buss C. Wadhwa P. (2012). Prenatal stress, telomere biology, and fetal programming of health and disease risk. Sci. Signal. 5, 12. 10.1126/scisignal.2003580 23112344

[B18] Ferezou-Viala J. Roy A. Serougne C. Gripois D. Parquet M. Bailleux V.. (2007). Long-term consequences of maternal high-fat feeding on hypothalamic leptin sensitivity and diet-induced obesity in the offspring. Am. J. Physiol. Regul. Integr. Comp. Physiol. 293 R1056–R1062. 10.1152/ajpregu.00117.2007 17553843

[B19] Fusco S. Ripoli C. Podda M. Chiatomone Ranieri S. Leone L. Toietta G.. (2012). A role for neuronal cAMP responsive-element binding (CREB)-1 in brain responses to calorie restriction. Proc. Natl. Acad. Sci. U.S.A. 109, 621–626. 10.1073/pnas.1109237109 22190495 PMC3258594

[B20] Garrido P. (2011). Aging and Stress: Past hypotheses, present approaches and perspectives. Aging Dis 2, 80–99. 22396868 PMC3295041

[B21] Gomez-Pinilla F. (2008). Brain foods: the effects of nutrients on brain function. Nat. Rev. Neurosci. 9, 568–578. 10.1038/nrn2421 18568016 PMC2805706

[B22] Harris R. (2000). Leptin - much more than a satiety signal. Annu. Rev. Nutr. 20, 45–75. 10.1146/annurev.nutr.20.1.45 10940326

[B23] Hood M. Moore L. Sundarajan-Ramamurti A. Singer M. Cupples L. Ellison R. (2000). Parental eating attitudes and the development of obesity in children: the Framingham Children's Study. Int. J. Obes. 24, 1319–1325. 10.1038/sj.ijo.0801396 11093294

[B24] Jirtle R. Skinner M. (2007). Environmental epigenomics and disease susceptibility. Nat. Rev. Genet. 8, 253–262. 10.1038/nrg2045 17363974 PMC5940010

[B25] Kalichman L. Rodrigues B. Gurvich D. Isrealov Z. Spivak E. (2007). Impact of patients weight on stroke rehabilitation results. Am. J. Phys. Med. Rehabil. 86, 650–655. 10.1097/PHM.0b013e318115f41b 17667195

[B26] Kaplan G. B. Vasterling J. Vedak P. (2010). Brain-derived neurotrophic factor in traumatic brain injury, post-traumatic stress disorder and their comorbid conditions: role in pathogenesis and treatment. Behav. Pharmacol. 21, 427–437. 10.1097/FBP.0b013e32833d8bc9 20679891

[B27] Kolb B. Whishaw I. (2008). Fundamentals of Human Neuropsychology, New York, NY, Worth Publishers.

[B28] Langdon K. Clarke J. Corbett D. (2011). Long-term exposure to high fat diet is bad for your brain: exacerbation of focal ischemic brain injury. Neuroscience 182, 82–87. 10.1016/j.neuroscience.2011.03.028 21435380

[B29] Lavu S. Boss O. Elliot P. J. Lambert P. (2008). D. Sirtuins - Novel therapuetic targets to treat age-associated diseases. Nat Rev. 7, 841–853. 10.1038/nrd2665 18827827

[B30] Lee J. Seroogy K. Mattson M. (2002). Dietary restriction enhances neurotrophin expression and neurogenesis in the hippocampus of adult mice. J. Neurochem. 80, 539–547. 10.1046/j.0022-3042.2001.00747.x 11905999

[B31] Li Y. Daniel M. Tollefsbol T. (2011). Epigenetic regulation of caloric restriction in aging. BMC Med. 9:98. 10.1186/1741-7015-9-98 21867551 PMC3175174

[B32] Lindqvist A. Mohapel P. Bouter B. Frielingsdorf H. Pizzo D. Brundin P. (2006). High-fat diet impairs hippocampal neurogenesis in rats. Eur. J. Neurol. 13, 1385–1388. 10.1111/j.1468-1331.2006.01500.x 17116226

[B33] Liu L. Lai S. Andrews L. Tollefsbol T. (2004). Genetic and epigenetic modultion of telomerase activity in development and disease. Gene 340, 1–10. 10.1016/j.gene.2004.06.011 15556289

[B34] Luchsinger J. Tang M. Shea S. Mayeux R. (2002). Caloric intake and the risk of Alzheimer disease. Arch. Neurol. 59, 1258–1263. 10.1001/archneur.59.8.1258 12164721

[B35] Masoro E. (2005). Overview of caloric restriction and ageing. Mech. Ageing Dev. 126, 913–911. 10.1016/j.mad.2005.03.012 15885745

[B36] Mattson M. (2000). Emerging neuroprotective strategies for Alzheimer's disease: dietary restriction, telomerase activation, and stem cell therapy. Exp. Gerontol. 35, 489–502. 10.1016/S0531-5565(00)00115-7 10959037

[B37] Mattson M. (2010). The impact of dietary energy intake on cognitive aging. Front. Aging Neurosci. 2:5. 10.3389/neuro.24.005.2010 20552045 PMC2874403

[B38] Mattson M. Duan W. Guo Z. (2003). Meal size and frequency affect neuronal plasticit and vulnerability to disease: cellular and molecular mechanisms. J. Neurochem. 84, 417–431. 10.1046/j.1471-4159.2003.01586.x 12558961

[B39] Max J. Lindgren S. Knutson C. Pearson C. Ihrig D. Welborn A. (1998). Child and adolescent traumatic brain injury: correlates of disruptive behavior disorders. Brain Inj. 12, 41–52. 10.1080/026990598122845 9483336

[B40] McCarthy M. Auger A. Bale T. De Vries G. Dunn G. Forger N.. (2009). The epigenetics of sex differences in the brain. J. Neuro 29, 12815–12823. 10.1523/JNEUROSCI.3331-09.2009 19828794 PMC2788155

[B41] McCrory P. Meeuwisse W. Aubry M. Cantu B. Dvorak J. Echemendia R.. (2013). Consensus statement on concussion in sport: the 4th International Conference on Concussion in Sport held in Zurich, November 2012. Br. J. Sports Med. 47, 250–258. 10.1136/bjsports-2013-092313 23479479

[B42] Molteni R. Barnard R. Ying Z. Roberts C. Gomez-Pinilla F. (2002). A high-fat, refined sugar diet reduces hippocampal brain-derived neurotrophic factor, neuronal plasticity, and learning. Neuroscience 112, 803–814. 10.1016/S0306-4522(02)00123-9 12088740

[B43] Molteni R. Fumagalli F. Magnaghi V. Roceri M. Gennarelli M. Racagni G.. (2001). Modulation of fibroblast growth factor-2 by stress and corticosteroids: from developmental events to adult brain plasticity. Brain Res. Rev. 37, 249–258. 10.1016/S0165-0173(01)00128-X 11744090

[B44] Mychasiuk R. Farran A. Esser M. (2014a). J. Assessment of an experimental rodent model of pediatric mild traumatic brain injury J. Neurotrauma 31, 1–9. 10.1089/neu.2013.3132 24283269

[B45] Mychasiuk R. Hehar H. Farran A. Esser M. J. (2014b). Mean Girls: sex differences in the effects of mild traumatic brain injury on the social dynamics of juvenile rat play behaviour. Behav. Brain Res. 259, 284–291. 10.1016/j.bbr.2013.10.048 24231261

[B46] Mychasiuk R. Hehar H. Ma I. Esser M. J. (2015). Dietary intake alters behavioural recovery and gene expression profiles in the brain of juvenile rats that have experienced a concussion. Front. Behav. Neurosci. 9:17 10.3389/fnbeh.2015.00017 PMC4318392 25698949

[B47] Paul L. (2011). Diet, nutrition, and telomere length. J. Nutr. Biochem. 22, 895–901. 10.1016/j.jnutbio.2010.12.001 21429730

[B48] Pfaffl M. (2001). A new mathematical model for relative quantification in real-time RT-PCR. Nucleic Acids Res. 29:e45. 10.1093/nar/29.9.e45 11328886 PMC55695

[B49] Pistell P. Morrison C. D. Gupta S. Knight A. Keller J. N. Ingram D. (2010). Cognitive impairment following high fat diet consumption is associated with brain inflamation. J. Neuroimmunol. 219, 25–32. 10.1016/j.jneuroim.2009.11.010 20004026 PMC2823983

[B50] Rabinovsky E. (2004). The multifunctional role of IGF-1 in peripheral nerve regeneration. Neurol. Res. 26, 204–210. 10.1179/016164104225013851 15072640

[B51] Rich N. J. VanLandingham J. W. Figueiroa S. Seth R. Corniola R. Levenson C. (2010). Chronic caloric restriction reduces tissue damage and improves spatial memory in a rat model of traumatic brain injury. J. Neurosci. Res. 88, 2933–2939. 10.1002/jnr.22443 20544832

[B52] Savage J. Orlet Fisher J. Birch L. (2007). Parenting influence on eating behavior. J Law Med. Ethics 35, 22–34. 10.1111/j.1748-720X.2007.00111.x 17341215 PMC2531152

[B53] Schallert T. Woodlee M. Fleming S. (2002). Disentangling multiple types of recovery from brain injury, in Pharmacology of Cerebral Ischemia, eds Krieglstein J. Klumpp S. (Stuttgart: Medpharm Scientific Publishers), 201–216.

[B54] Sharma S. Kaur G. (2005). Neuroprotective potential of dietary restriction against kainate-induced excitotxicity in adult male rats. Brain Res. Bull. 67, 482–491. 10.1016/j.brainresbull.2005.07.015 16216697

[B55] Spanswick S. Sutherland R. (2010). Object/context-specific memory deficits associated with loss of hippocampal granule cells after adrenalectomy in rats. Learn. Mem. 17, 241–245. 10.1101/lm.1746710 20410060 PMC2893217

[B56] Stranahan A. Norman G. Lee K. Cutler R. Telijohann R. Egan J.. (2008). Diet-induced insulin resistence impairs hippocampal synaptic placticity and cognition in middle aged rats. Hippocampus 18, 1085–1088. 10.1002/hipo.20470 18651634 PMC2694409

[B57] Suk S. Sacco R. Boden-Albala B. Cheun J. Pittman J. Elkind M.. (2003). Abdominal obesity and risk of ischemic stroke. Stroke 34, 1586–1592. 10.1161/01.STR.0000075294.98582.2F 12775882

[B58] Sutherland R. Whishaw I. Kolb B. (1988). Contributions of cingulate cortex to two forms of spatial learning and memory. J. Neuro 8, 1863–1872. 3385478 10.1523/JNEUROSCI.08-06-01863.1988 PMC6569344

[B59] Tatebayashi Y. Lee M. H. Iqbal K. Grundke-Iqbal I. (2003). The dentate gyrus neurogenesis: a therapeutic target for Alzheimer's disease. Acta Neuropathol. (Berl.) 105, 225–232. 10.1007/s00401-002-0636-3 12557008

[B60] Valdes A. Andrew T. Gardner J. Kimura M. Oelsner E. Cherkas L.. (2005). Obesity, cigaretty smoking, and telomere length in women. Lancet 366, 662–664. 10.1016/S0140-6736(05)66630-5 16112303

[B61] Vera E. Bernardes de Jesus B. Foronda M. Flores J. Blasco M. (2013). Telomerase reverse transcriptase synergizes with calorie restriction to increase health span and extend mouse longevity. PLoS ONE 8:e53760. 10.1371/journal.pone.0053760 23349740 PMC3551964

[B62] Wang J. Ho L. Weiping Q. Rocher A. Seror I. Humala N.. (2005). Caloric restriction attenuates B-amyloid neuropathology in a mouse model of Alzheimer's disease. FASEB J. 19, 659–661. 15650008 10.1096/fj.04-3182fje

[B63] Weindruch R. Sohal R. (1997). Caloric intake and aging. N.Engl. J. Med. 337, 986–994. 10.1056/NEJM199710023371407 9309105 PMC2851235

[B64] Witte A. Fobker M. Gellner R. Knecht S. Floel A. (2009). Caloric restriction improves memory in elderly humans. Proc. Natl. Acad. Sci. U.S.A. 106, 1255–1260. 10.1073/pnas.0808587106 19171901 PMC2633586

[B65] Wu A. Molteni R. Ying Z. Gomez-Pinilla F. (2003). A saturated-fat diet aggrevates the outcome of traumatic brain injury on hippocampal plasticity and cognitive function by reducing brain-derived neurotrophic factors. Neuroscience 119, 365–375. 10.1016/S0306-4522(03)00154-4 12770552

[B66] Wu A. Ying Z. Gomez-Pinilla F. (2004). The interplay between oxidative stress and brain-derived neurotrophic factor modulates the outcome of a saturated fat diet on synaptic plasticity and cognition. Eur. J. Neurosci. 19, 1699–1707. 10.1111/j.1460-9568.2004.03246.x 15078544

[B67] Wu M. Manoli D. Fraser E. Coats K. Tollkuhn J. Honda S.. (2009). Estrogen masculinizes neural pathways and sex-specific behaviors. Cell 139, 61–72. 10.1016/j.cell.2009.07.036 19804754 PMC2851224

[B68] Zilles K. (1985). The Cortex of the Rat: A Stereotaxis Atlas. Berlin, Springer-Verlag.

